# Cyclotron production and radiochemical purification of terbium-155 for SPECT imaging

**DOI:** 10.1186/s41181-021-00153-w

**Published:** 2021-11-14

**Authors:** C. Favaretto, Z. Talip, F. Borgna, P. V. Grundler, G. Dellepiane, A. Sommerhalder, H. Zhang, R. Schibli, S. Braccini, C. Müller, N. P. van der Meulen

**Affiliations:** 1 Center for Radiopharmaceutical Sciences, ETH-PSI-USZ, Paul Scherrer Institute, 5232 Villigen-PSI, Switzerland; 2 Albert Einstein Center for Fundamental Physics (AEC), Laboratory of High Energy Physics (LHEP), University of Bern, 3012 Bern, Switzerland; 3 Division Large Research Facilities, Paul Scherrer Institute, 5232 Villigen-PSI, Switzerland; 4 Department of Chemistry and Applied Biosciences, ETH Zurich, 8093 Zurich, Switzerland; 5 Laboratory of Radiochemistry, Paul Scherrer Institute, 5232 Villigen-PSI, Switzerland

**Keywords:** Terbium-155, Matched pairs, Radiolanthanides, Theragnostics, Radionuclide production, Proton irradiation, Cyclotron, Radiochemical purification, SPECT/CT imaging

## Abstract

**Background:**

Terbium-155 [T_1/2_ = 5.32 d, Eγ = 87 keV (32%) 105 keV (25%)] is an interesting radionuclide suitable for single photon emission computed tomography (SPECT) imaging with potential application in the diagnosis of oncological disease. It shows similar decay characteristics to the clinically established indium-111 and would be a useful substitute for the diagnosis and prospective dosimetry with biomolecules that are afterwards labeled with therapeutic radiolanthanides and pseudo-radiolanthanides, such as lutetium-177 and yttrium-90. Moreover, terbium-155 could form part of the perfect “matched pair” with the therapeutic radionuclide terbium-161, making the concept of true radiotheragnostics a reality. The aim of this study was the investigation of the production of terbium-155 via the ^155^Gd(p,n)^155^Tb and ^156^Gd(p,2n)^155^Tb nuclear reactions and its subsequent purification, in order to obtain a final product in quantity and quality sufficient for preclinical application. The ^156^Gd(p,2n)^155^Tb nuclear reaction was performed with 72 MeV protons (degraded to ~ 23 MeV), while the ^155^Gd(p,n)^155^Tb reaction was degraded further to ~ 10 MeV, as well as performed at an 18 MeV medical cyclotron, to demonstrate its feasibility of production.

**Result:**

The ^156^Gd(p,2n)^155^Tb nuclear reaction demonstrated higher production yields of up to 1.7 GBq, however, lower radionuclidic purity when compared to the final product (~ 200 MBq) of the ^155^Gd(p,n)^155^Tb nuclear reaction. In particular, other radioisotopes of terbium were produced as side products. The radiochemical purification of terbium-155 from the target material was developed to provide up to 1.0 GBq product in a small volume (~ 1 mL 0.05 M HCl), suitable for radiolabeling purposes. The high chemical purity of terbium-155 was proven by radiolabeling experiments at molar activities up to 100 MBq/nmol. SPECT/CT experiments were performed in tumor-bearing mice using [^155^Tb]Tb-DOTATOC.

**Conclusion:**

This study demonstrated two possible production routes for high activities of terbium-155 using a cyclotron, indicating that the radionuclide is more accessible than the exclusive mass-separated method previously demonstrated. The developed radiochemical purification of terbium-155 from the target material yielded [^155^Tb]TbCl_3_ in high chemical purity. As a result, initial cell uptake investigations, as well as SPECT/CT in vivo studies with [^155^Tb]Tb-DOTATOC, were successfully performed, indicating that the chemical separation produced a product with suitable quality for preclinical studies.

**Supplementary Information:**

The online version contains supplementary material available at 10.1186/s41181-021-00153-w.

## Background

In recent years, nuclear medicine emerged as an increasingly important area in the diagnosis and treatment of both oncological and non-oncological diseases (Weber et al. 2020). In particular, in the field of diagnosis, Single Photon Emission Computed Tomography (SPECT) has been used for many years as a non-invasive imaging technique. (Mariani et al. 2010). The combination of SPECT with CT increased interest in the technique for its accuracy in the depiction of the localization of disease (Buck et al. 2008). This application, however, demands radionuclides with the appropriate decay properties. To date, SPECT/CT had applications and expansion mainly thanks to the use of technetium-99 m [Eγ = 141 keV (89%), T_1/2_ = 6.01 h, (Browne and Tuli 2017)] which is the most often used radionuclide in clinical diagnostic nuclear medicine (Lee 2019).

Our group is investigating the emerging role of novel promising radionuclides for theragnostics (Domnanich et al. 2016; Gracheva et al. 2019; van der Meulen et al. 2015, 2020b; Müller et al. 2012). Among these radionuclides, terbium-155 is of high interest because it is a radiolanthanide that emits γ-rays of energies suitable for SPECT [Eγ = 87 keV (32%) 105 keV (25%)] (Nica 2019). Terbium-155 decays with a half-life of 5.32 days, which can allow the study of pre-therapeutic dosimetry and tumor visualization at later time points after administration (Müller et al. 2014a). The mentioned decay properties make terbium-155 a valid alternative to indium-111 [Eγ = 245 keV (94%) 171 keV (91%), T_1/2_ = 2.80 d, (Blachot 2009)], which has been employed for the diagnosis and pretreatment dosimetry with biomolecules that are afterwards labeled with radiolanthanides and pseudo-radiolanthanides, such as lutetium-177 and yttrium-90, commonly used for β^−^-particle therapy (Witzig 2001). It is worth mentioning, however, that the small differences in the coordination chemistry between indium and lanthanides may lead to differences in the biodistribution of the correspondingly-radiolabeled compound (Camera et al. 1994). Therefore, indium-111 is not a perfect diagnostic match to therapeutic radiolanthanides. The radiolanthanide terbium-155 would enable using chemically identical or nearly identical radiopharmaceuticals for imaging and therapy and, thus, allow its application for prospective dosimetry (Borgna et al. 2021). Furthermore, the optimal practice would be the combined use of the same molecular targeting vectors, labeled either with a diagnostic or therapeutic radionuclide of the same element. This approach is known as radiotheragnostics and would allow the most accurate proceeding for the diagnosis and dosimetry on the patient before performing the treatment (Rösch et al. 2017). In this regard, terbium-155 may be used as a precise diagnostic match for the α-emitter terbium-149 (Müller et al. 2012; Umbricht et al. 2019) or, in a greater degree, for the β^−^-particle-emitting terbium-161 (T_1/2_ = 6.96 days, (Durán et al. 2020)), which has recently been produced in high quality and tested in preclinical and clinical studies (Baum et al. 2021; Favaretto et al. 2021; Gracheva et al. 2019; Müller et al. 2019). Moreover, the long half-life of terbium-155 would be favorable for tumor visualization at late time points after injection which may be interesting in particular with longer-circulating biomolecules. Therefore, the availability of large quantities of terbium-155 with adequate purity would be of particular value for clinical application.

**Fig. 1:**
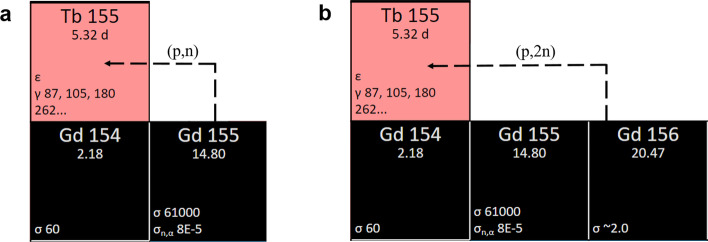
Nuclear reactions for the production of terbium-155 via proton irradiation of enriched [^155^Gd]Gd_2_O_3_ and [^156^Gd]Gd_2_O_3_ targets. ^155^Gd(p,n)^155^Tb (**a**) and ^156^Gd(p,2n)^155^Tb (**b**).

To date, previous studies reported terbium-155 production via high-energy proton-induced spallation of a tantalum target followed by ionization and mass separation (Fiaccabrino et al. 2021; Müller et al. 2012, 2014a; Webster et al. 2019). The current limitation of this method is the overall separation efficiency due to the currently poor efficiency of surface ionization, thus, the production of terbium-155 in large quantities via this production route appears not to be a viable option. Other than the spallation reactions, the production of terbium-155 is also possible by means of two proton-induced direct reactions: ^155^Gd(p,n)^155^Tb and ^156^Gd(p,2n)^155^Tb (Fig. 1) (Dmitriev et al. 1989). The accumulation of terbium radionuclide impurities is sensibly reduced, thanks to the use of highly-enriched gadolinium oxide as target material that results in the production of high activities of terbium-155 with minimal radionuclidic contaminants (Vermeulen et al. 2012). Cross section measurements using these target materials were performed and will be reported elsewhere (Dellepiane et al. 2022). The ^156^Gd(p,2n)^155^Tb nuclear reaction suggests high production yields, however, it requires the use of higher-energy cyclotrons. The ^155^Gd(p,n)^155^Tb reaction can be performed with medical cyclotrons (typical proton energy, 18 MeV), allowing the production of terbium-155 with such a device.

The aim of the present study was to explore, for the first time, the production of high terbium-155 activities via proton irradiation of highly-enriched gadolinium-155 and gadolinium-156 oxide targets at Paul Scherrer Institute (PSI), Switzerland, and its subsequent radiochemical separation from target material and impurities. Terbium-155 labeling experiments were performed as part of the quality control, and the utility of terbium-155 for SPECT imaging demonstrated.

## Methods

### Terbium-155 production

#### Target preparation

Enriched gadolinium oxide ([^155^Gd]Gd_2_O_3_ 91.9% enrichment or [^156^Gd]Gd_2_O_3_ 93.3% enrichment, Isoflex, USA) was used as target material for the production of terbium-155 via the ^155^Gd(p,n)^155^Tb and ^156^Gd(p,2n)^155^Tb nuclear reactions, respectively. The elemental composition of the target materials in question is provided in Additional file 1 (Additional file 1: Tables S1 and S2). Approximately 40 mg of material was pressed into ~ 6 mm diameter disc-shaped pellets, 0.3 mm thick, by applying an axial pressure of 2 tons for 5 s.

#### Target irradiations using PSI’s Injector 2 cyclotron

**Fig. 2:**
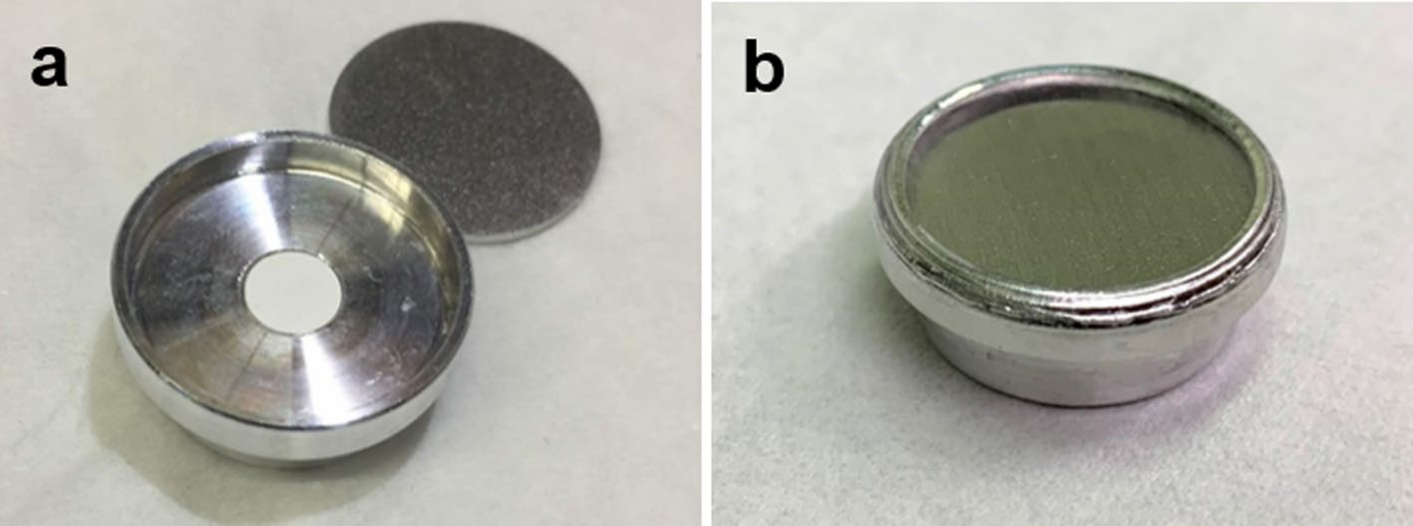
Open (**a**) target capsule containing a 6mm gadolinium oxide target nestled in an indentation, and (**b**) sealed target capsule

The pellets produced by compression of the enriched material were encapsulated in aluminum (99.5% pure) capsules specifically designed in-house for PSI’s IP2 irradiation station (Grundler et al. 2020; van der Meulen et al. 2020a) (Fig. 2).

The gadolinium targets were irradiated, using the 72 MeV proton beam from the Injector 2 separated sector cyclotron. The beam current was set to 50 µA. Niobium discs (3.4 mm and 2.4 mm, respectively) were used as degraders to decrease the proton energy from 72 MeV to the desired energy windows (Grundler et al. 2020; van der Meulen et al. 2015, 2020a). The thicknesses of the degraders were calculated using SRIM-2013 (Ziegler et al. 2010). The beam energy was set to ~ 10.3 MeV for the (p,n) reaction and ~ 22.8 MeV for the (p,2n) reaction according to the cross-section measurements previously performed (Dellepiane et al. 2022; Dmitriev et al. 1989; Vermeulen et al. 2012). The irradiations were performed for up to 8 h (Additional file 1: Table S3). The energy of the protons exiting the target was calculated using SRIM for the case of 40 mg targets. It was found to be (20.7 ± 1.5) MeV and (6.0 ± 3.4) MeV, for entry energies of 22.8 MeV and 10.3 MeV, respectively.

#### Target irradiations at the Bern medical cyclotron

**Fig. 3:**
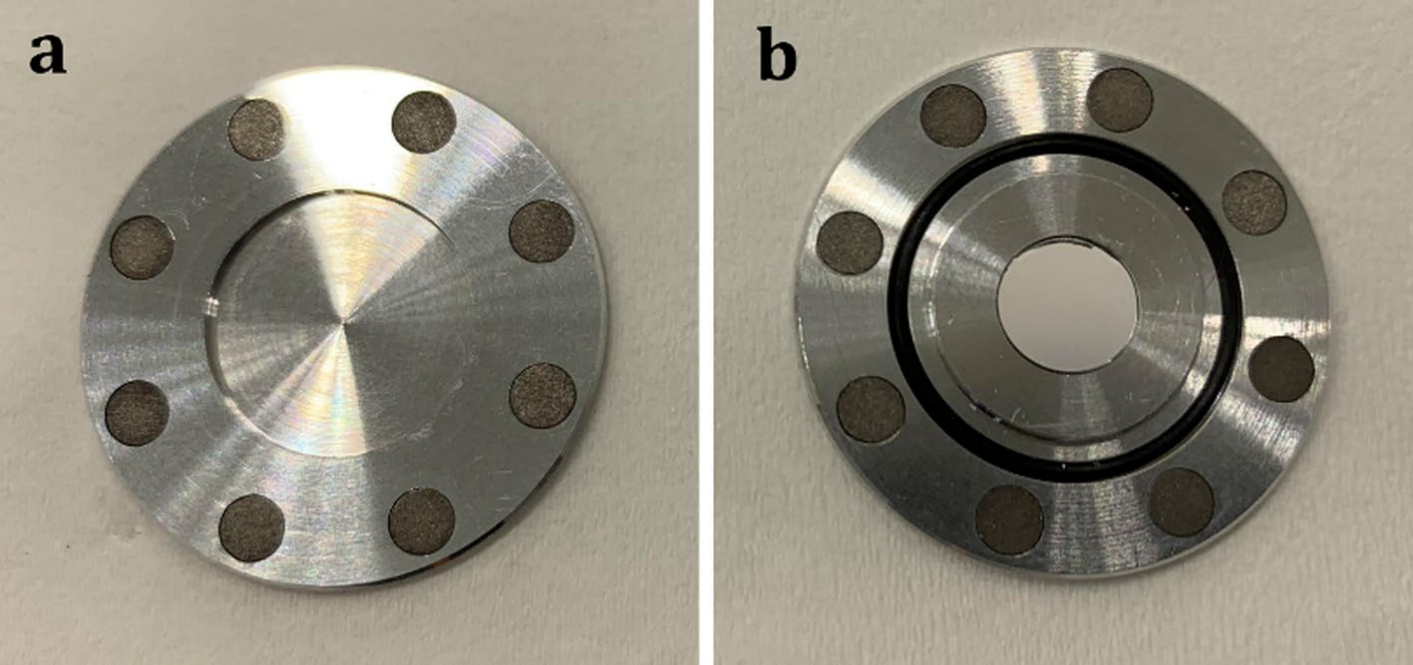
Covering lid (**a**) and back part (**b**) of the “coin”, containing a 6mm gadolinium oxide target.

Two irradiations were performed with the medical cyclotron (IBA Cyclone HC 18 MeV) in operation at the Bern University Hospital (Inselspital), to demonstrate the feasibility to produce terbium-155 via the ^155^Gd(p,n)^155^Tb nuclear reaction with such a machine. This facility features two bunkers with independent access, the former for the cyclotron and the latter for a 6 m long Beam Transfer Line (BTL) for multidisciplinary research activities (Braccini 2013). The cyclotron is equipped with an IBA Nirta Solid Target Station (STS), a mechanical transfer system (named Hyperloop) to load the STS from outside the cyclotron bunker and a pneumatic solid target transfer system (STTS) by Tema Sinergie to deliver the irradiated target either to a hot-cell or to a receiving station located in the BTL bunker (Braccini et al. 2020). To assess the produced activity, a semiconductor detector system based on a 3 cm^3^ CdZnTe (CZT) crystal was installed in the receiving station (Dellepiane et al. 2021). The [^155^Gd]Gd_2_O_3_ targets for the irradiation at the medical cyclotron were prepared by compression of enriched material, as described above. The disc-shaped pellets were inserted in a specific capsule—named “coin”—composed by two halves kept together by permanent magnets, as shown in Fig. 3. This capsule was developed by the Laboratory of High Energy Physics (LHEP) at the University of Bern, Switzerland, and was previously successfully used to produce scandium-44 (van der Meulen et al. 2020b).

The two irradiations of the [^155^Gd]Gd_2_O_3_ targets were performed at 2.4 µA and 0.7 µA beam intensity with 10.9 MeV and 10.5 MeV proton energy, respectively (Additional file 1: Table S4). The entry and exit energies were tuned by adjusting the thickness of the covering lid of the coin. On the basis of the measured production cross sections, the energies were chosen to optimize the production yield, while minimizing the impurities (Dellepiane et al. 2022). The irradiations were conducted for 70 and 40 min, respectively.

### Radiochemical separation process

The terbium-155 separation from the gadolinium target material and impurities was developed by means of bench experiments using three types of resin (described below) with the use of γ-emitting radioactive tracers (gadolinium-153 and terbium-160) and ICP-standards of the elements of interest (Additional file 1). After loading the columns, the lanthanides were eluted with suitable eluents (see below), and the fractions obtained from the elutions measured by means of γ-ray spectrometry or ICP-OES (Agilent 5110 ICP-OES, USA). With the data gathered from these experiments, the elution profiles of terbium and gadolinium from three different types of resins were established (Additional file 1: Figs. S1–S5).

Based on the bench experiments results, a chemical separation system was designed, built, and introduced into a hot cell, such that high activities of the radionuclide could be manipulated with reduced radiation-dose exposure to the operator. Selected irradiated targets (excluding production runs 1 and 4, that were performed only for production yield data) were processed using this setup. After the irradiation, the targets were removed from their aluminum capsules in a hot cell, placed into a Reacti-Vial™ (Thermo Fisher Scientific, USA) and transferred to the separation panel. The target was dissolved in 5 mL 7.0 M HNO_3_ (Suprapur, Merck, Germany) by heating for 30 min at 100 °C. The solution was loaded onto a chromatographic column (10 mm × 100 mm) filled with *N,N,N’,N’*-tetra-n-octyldiglycolamide extraction resin (DGA Normal resin, Triskem International, France; particle size 50–100 μm, column volume: 8.5 mL). Terbium-155 elution from the DGA column was performed with the use of 0.05 M HCl (Suprapur, Merck, Germany) as eluent (0.75 mL/min flow rate). While the first 15 mL of elution were discarded, the following 25 mL were loaded onto a column containing Sykam macroporous cation exchange resin (Sykam Chromatographie Vertriebs GmbH, Germany; particle size 12–22 μm, NH_4_^+^ form, column volume: 19.8 mL) followed by 60 mL Milli-Q water. The terbium-155 separation from the target material was performed on the Sykam column with the use of ~ 100 mL 0.13 M (pH 4.5) α-hydroxy-isobutyric acid (α-HIBA, Sigma-Aldrich GmbH, Germany) as eluent (0.6 mL/min flow rate). The eluted fraction containing terbium-155 (~ 25 mL 0.13 M α-HIBA) was loaded onto the last column of the separation panel, prepared using ~ 0.08 mL of bis(2,4,4-trimethyl-1-pentyl)phosphinic acid extraction resin (LN3, Triskem International, France; particle size 50–100 μm) slurried in a 1 mL column. The final purification was performed by rinsing the LN3 resin with 10 mL Milli-Q water followed by 14 mL 0.01 M HCl (Suprapur, Merck GmbH, Germany) at 1 mL/min. Finally, terbium-155 was eluted in a small volume (~ 1 mL 0.05 M HCl) at a flow rate of 0.1 mL/min in the form of [^155^Tb]TbCl_3_. During the entire process, the movement of terbium-155 activity along the column was monitored by means of pin diodes, which acted as detectors. The pH of the final product was determined using pH indicator strips. The target material separated from terbium-155 in each purification process could be recovered and recycled for reuse in future irradiations and productions (Additional file 1).

### Production yield, separation yield and radionuclidic purity

The terbium-155 activities were determined before and after separation by γ-ray spectrometry using a high-purity germanium (HPGe) detector (Canberra, France), in combination with the Inter-Winner software package (version 7.1, Itech Instruments, France). The efficiency calibration was performed using an Eppendorf vial filled with a europium-152 solution (89.51 kBq ± 0.71%, reference date 20.02.2017) and placed at 1 m from the detector. A 5 µL aliquot of the final product ([^155^Tb]TbCl_3_) was introduced in an Eppendorf vial and measured with uncertainty ≤ 5% at 1 m from the detector. Moreover, a small aliquot of the solution obtained from the target dissolution (5 mL 7.0 M HNO_3_) was also measured with the same method. As a result, it was possible to calculate the production and separation yields, by decay correction and sample extrapolation, together with the activity obtained at the end of bombardment (EOB), and at the end of separation (EOS), respectively. The radionuclidic purity at EOS was assessed using the same method.

### Radiolabeling yield

After every separation process, the radiolabeling yield of the terbium-155 product was assessed to indirectly evaluate the chemical purity of the product and to ensure the capability of the radionuclide to radiolabel biomolecules at high molar activities required for (pre)clinical use. Terbium-155 (~ 100 MBq) was added to a reaction mixture consisting of 0.05 M HCl and 0.5 M sodium acetate with a final pH of ~ 4.5. DOTATOC (1 mM) was added to obtain molar activities up to 100 MBq/nmol. The reaction mixture was incubated at 95 °C for 10 min (Müller et al. 2014b). Quality control was performed to determine the radiolabeled fractions of DOTATOC, as previously reported, by means of high-performance liquid chromatography (HPLC, Merck Hitachi, LaChrom) (Additional file 1) (Gracheva et al. 2019).

The radiolabeled samples with low radiolabeling yields were further analyzed by means of liquid chromatography-mass spectrometry-electrospray ionization (LC-MS-ESI, Waters LCT Premier mass spectrometer) to identify the present metals (impurities) labeled to the peptide (Additional file 1).

### Preclinical studies

Cell internalization and uptake of the ^155^Tb-labeled somatostatin (SST) analogue DOTATOC were determined in somatostatin receptor (SSTR)-positive AR42J cells, according to a previously-published procedure (Additional file 1) (Borgna et al. 2021).

Animal studies were ethically approved by the responsible Committee of Animal Experimentation and permitted by the responsible cantonal authorities (license No. 75721) (Additional file 1). Five-week-old female, CD-1 nude mice (Charles River Laboratories, Germany) were subcutaneously inoculated with AR42J tumor cells (5 × 10^6^ cells in 100 µL PBS) as previously reported (Borgna et al. 2021). The SPECT/CT study was performed 10–14 days after tumor cell inoculation when the tumor size reached a volume of ~ 250 mm^3^.

### SPECT/CT imaging studies

A small-animal, 4-head multiplexing, multipinhole camera (NanoSPECT/CT; Mediso Medical Imaging Systems, Budapest, Hungary) was employed for SPECT/CT imaging studies, as previously reported (Additional file 1) (Borgna et al. 2021). In brief, the SPECT scans were acquired using Nucline software (version 1.02, Mediso Ltd., Budapest, Hungary) using energy windows set at 46.0 keV (± 8.5%), 86.0 keV (± 8.5%) and 105.0 keV (± 10%). The real-time CT reconstruction used a cone-beam filtered backprojection. SPECT data were reconstructed iteratively with HiSPECT software (version 1.4.3049, Scivis GmbH, Göttingen, Germany) using standard settings. SPECT and CT data were automatically co-registered because both modalities shared the same axis of rotation. The fused datasets were processed using VivoQuant (version 3.5, inviCRO Imaging Services and Software, Boston USA). A Gaussian post-reconstruction filter (FWHM = 1.0 mm) was applied.

#### Derenzo phantom

A Derenzo phantom with a diameter of 19.5 mm, a height of 15.0 mm and with hole diameters ranging from 0.8 to 1.3 mm in 0.1-mm steps, was filled with approximately 10 MBq [^155^Tb]TbCl_3_ solution in a total volume of 900 µL containing 20% ethanol, to allow the complete filling of the capillaries (Bunka et al. 2016). Fifteen-minute SPECT/CT scans were performed with 240 s time-per-view and processed as described in the previous section.

#### In vivo studies

[^155^Tb]Tb-DOTATOC, prepared as described above for quality control, was diluted in 0.05% bovine serum albumin in PBS and intravenously injected in one AR42J-tumor-bearing mouse (~ 10 MBq, ~ 1 nmol). Scans were acquired 1, 4 and 24 h after the injection and, during the scans, the mouse was anesthetized by inhalation of an isoflurane–oxygen mixture. Time-per-view of the scans was set to 30 s, resulting in a scan time of about 20 min.

## Results

### Terbium-155 production

#### Production yields at PSI’s Injector 2 cyclotron

In agreement with the experimental cross section measurements (Dellepiane et al. 2022; Dmitriev et al. 1989), the (p,2n) reaction yielded higher activities compared to the (p,n) reaction. In particular, with 8 h irradiation of a [^155^Gd]Gd_2_O_3_ target, ~ 200 MBq terbium-155 was produced, while the same irradiation time on [^156^Gd]Gd_2_O_3_ targets yielded activities up to 1.7 GBq (Table 1, Additional file 1: Table S3).

**Table 1:** Terbium-155 activities produced via ^155^Gd(p,n)^155^Tb and ^156^Gd(p,2n)^155^Tb nuclear reactions at PSI’s IP 2 irradiation station and Bern medical cyclotron

Nuclear reaction	Facility	Target mass (mg)	Beam entry energy (MeV)	[^155^Tb]Tb EOB [MBq/(µAh)]
^155^Gd(p,n)^155^Tb	Bern Inselspital medical cyclotron	38.0	~ 10.7	3.09 ± 0.48
^155^Gd(p,n)^155^Tb	PSI injector 2	39.7 ± 1.2	~ 10.3	0.42 ± 0.26
^156^Gd(p,2n)^155^Tb	PSI injector 2	39.5 ± 0.8	~ 22.8	3.28 ± 0.65

#### Production yields at the Bern medical cyclotron

The irradiations performed at the Bern medical cyclotron yielded 7.7 ± 0.6 MBq and 1.6 ± 0.1 MBq of terbium-155, according to the irradiation conditions reported in Table 1 and Additional file 1: Table S4. The obtained activities were measured first with the CZT detector right after the irradiation and with an HPGe detector several days after the EOB. A good agreement was found between the two measurements and with the calculation of the produced activities based on the measured cross sections (Dellepiane et al. 2022).

### Radiochemical purification process

**Fig. 4:**
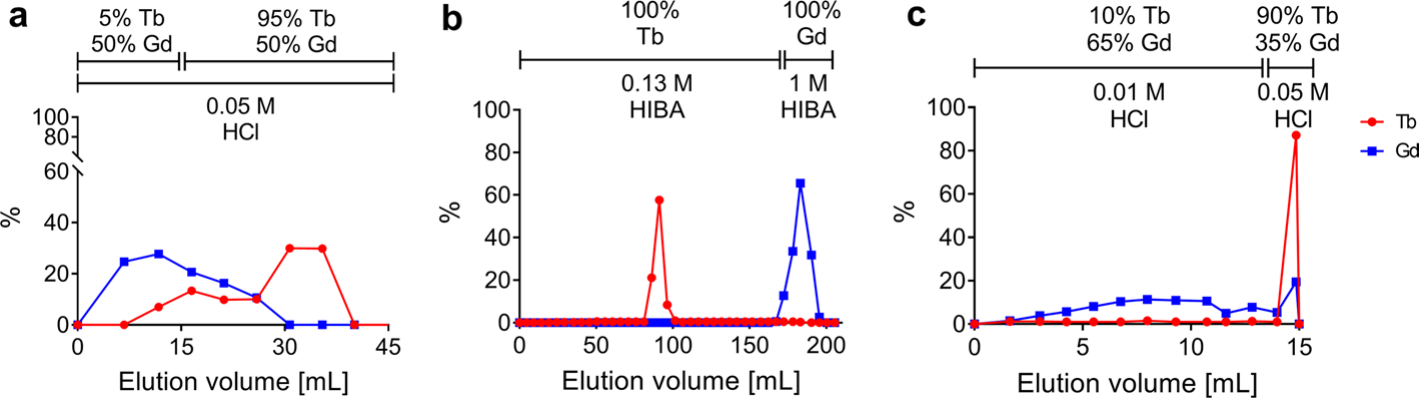
Representative elution profiles of terbium and gadolinium from DGA column using 0.05 M HCl (**a**), from Sykam column using 0.13 M and 1 M α-HIBA (**b**) and from LN3 column using 0.01 M HCl and 0.05 M HCl (**c**). In this set of experiments, the columns were loaded with lanthanide metal tracers, which were subsequently eluted with the eluents mentioned. The sample aliquots were measured by means of ICP-OES.

With the data gathered in bench experiments with stable isotopes, the elution profiles of terbium and gadolinium were established for the three columns proposed for the purification process (Fig. 4). The elution profile experiments performed with the DGA column showed an advantageous initial separation between terbium and gadolinium (Fig. 4a). In particular, ~ 50% of gadolinium and only ~ 5% of terbium were eluted with the first 15 mL of eluent while, with the following 25 mL, the remaining ~ 95% of terbium and ~ 50% of gadolinium could be removed from the column, resulting in a gadolinium/terbium separation factor of 2.00 ± 0.07%. Loading only 50% of the target material onto the Sykam column increased the resin purification performances tremendously (Additional file 1: Fig. S3) and resulted in two highly resolute peaks in the Sykam elution profile (Fig. 4b). With the experiments performed on the LN3 column (Additional file 1: Fig. S4 and S5), it was feasible to obtain a further separation between terbium and gadolinium (separation factor from ~ 4.5 to ~ 2.6, depending on flow rate) with a rinse step performed with 0.01 M HCl before the final elution step carried out with the higher concentrated acid (0.05 M HCl) (Fig. 4c).

**Fig. 5:**
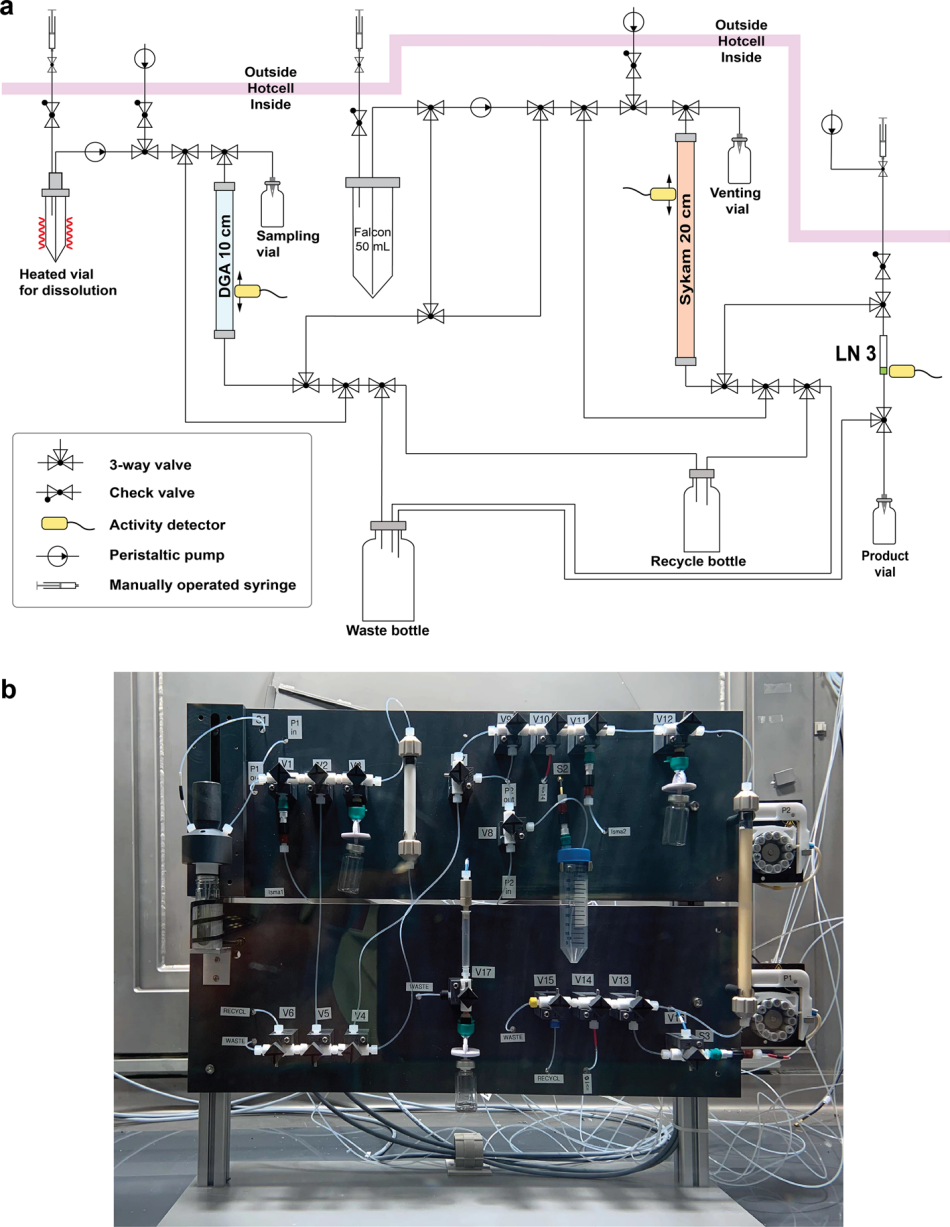
Schematic diagram of the terbium-155 separation panel (**a**) and image of the actual system (**b**). (Blue, orange and white colors in the diagram represent the columns that contain DGA, Sykam and LN3 resins, respectively).

A chemical separation system for the separation of terbium-155 from proton-irradiated enriched gadolinium oxide targets, and potential impurities, was designed and built based on the bench experiments (Fig. 5). After the irradiation, selected targets were dissolved and successfully processed through the three columns mentioned above and, finally, terbium-155 was eluted in 1.0 mL 0.05 M HCl (pH 1 – 2). A maximum of 7 h was needed to complete the radiochemical purification, thus, the process could be performed in one day.

### Separation yield and radionuclidic purity

The final product ([^155^Tb]TbCl_3_) in 1 mL 0.05 M HCl had an activity concentration of 0.02–1.0 MBq/μL. When performing the 500 µL-fractionated collection, however, it was determined that the first 500 μL fraction contained about 70 – 80% of the total activity.

The separation yields, calculated considering the initial terbium-155 activity of the targets after irradiation (EOB reported also in Additional file 1: Table S3 and S4), were estimated up to 93% (Table 2).

**Table 2:** Selected terbium-155 production runs, indicating measured activity EOB and EOS for each run

Production No	Nuclear reaction	Irradiation time (h)	[^155^Tb]Tb EOB (MBq)	[^155^Tb]Tb EOS (MBq)	[^155^Tb]Tb EOS (MBq) (corrected to EOB)	Separation yield (%)
2	^155^Gd(p,n)^155^Tb	4	26.8	18.1	23.7	88.4
5	^156^Gd(p,2n)^155^Tb	4	705	487	555	78.7
7	^156^Gd(p,2n)^155^Tb	8	1250	829	1029	82.3
8	^156^Gd(p,2n)^155^Tb	8	1684	1021	1279	75.9
9	^156^Gd(p,2n)^155^Tb	8	900	668	836	92.9

Productions 3 and 6 were excluded due to technical failures of the separation panel. To calculate the separation yield, the EOS activity was corrected to EOB time considering the decay correction (sixth column) and then used to calculate the fraction of initial activity recovered after the separation

The γ-ray spectrum obtained showed the γ-lines of terbium-155 together with those of terbium-156, terbium-154, terbium-154m and terbium-154m2 [Additional file 1: Table S5–S6 and Fig. S6, (Nica 2019; Reich 2009, Reich 2012)]. While terbium-156 has a half-life (T_1/2_ = 5.3 d) similar to terbium-155, the other radionuclidic impurities terbium-154, terbium-154m and terbium-154m2 are short-lived terbium isotopes with 21. 5 h, 9.4 h and 22.7 h half-lives, respectively [Additional file 1: Table S5, (Nica 2019; Reich 2009, Reich 2012)]. In particular, the measurements took place after each separation, approximately 2 days after EOB (Additional file 1: Table S6).

### Radiolabeling yield

Radiolabeling of DOTATOC with terbium-155 was reproducible at a molar activity of 50 MBq/nmol, with > 99% radiochemical yield. Depending on the activity concentration of the [^155^Tb]TbCl_3_ solution, it was feasible to label at higher molar activities of up to 100 MBq/nmol with > 99% radiochemical purity (Table 3 and Additional file 1: Fig. S7). The LC–MS-ESI analysis of the radiolabeled samples with radiolabeling yield < 95% showed the presence of [^155^Tb]Tb-DOTATOC together with Zn-DOTATOC and Fe-DOTATOC, while Gd-DOTATOC was not detected (Additional file 1: Fig. S8 for radiolabeling of production 9 at 100 MBq/nmol).

**Table 3:** Quality control of terbium-155 after the radiochemical separation process (the molar activity of radiolabeled product)

Production no	Nuclear reaction	Irradiation time (h)	[^155^Tb]Tb EOS (MBq)	> 95% radiolabeled molar activity (MBq/nmol)
2	^155^Gd(p,n)^155^Tb	4	18.1	n.a
5	^156^Gd(p,2n)^155^Tb	4	487	100
7	^156^Gd(p,2n)^155^Tb	8	829	100
8	^156^Gd(p,2n)^155^Tb	8	1021	100
9	^156^Gd(p,2n)^155^Tb	8	668	50

### SPECT/CT imaging studies

#### Derenzo phantom

**Fig. 6:**
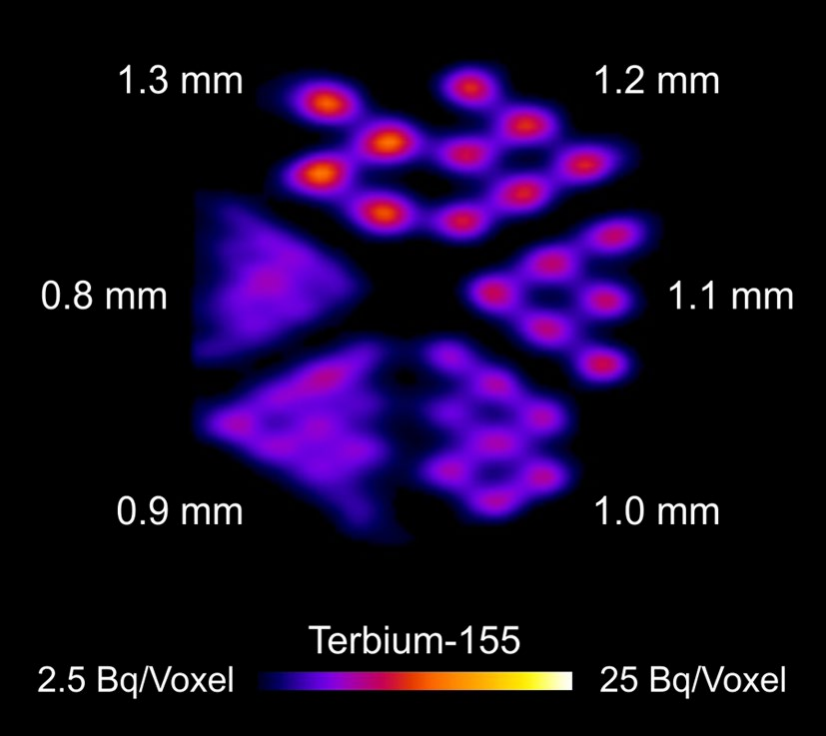
Fifteen-minute SPECT scan of sagittal sections of Derenzo phantom filled with terbium-155 (10 MBq). No filter was applied.

Derenzo phantom studies performed with terbium-155 showed excellent spatial resolution and resolvable line widths up to 1.0 mm after 15 min scanning time (Fig. 6).

#### In vivo studies

**Fig. 7:**
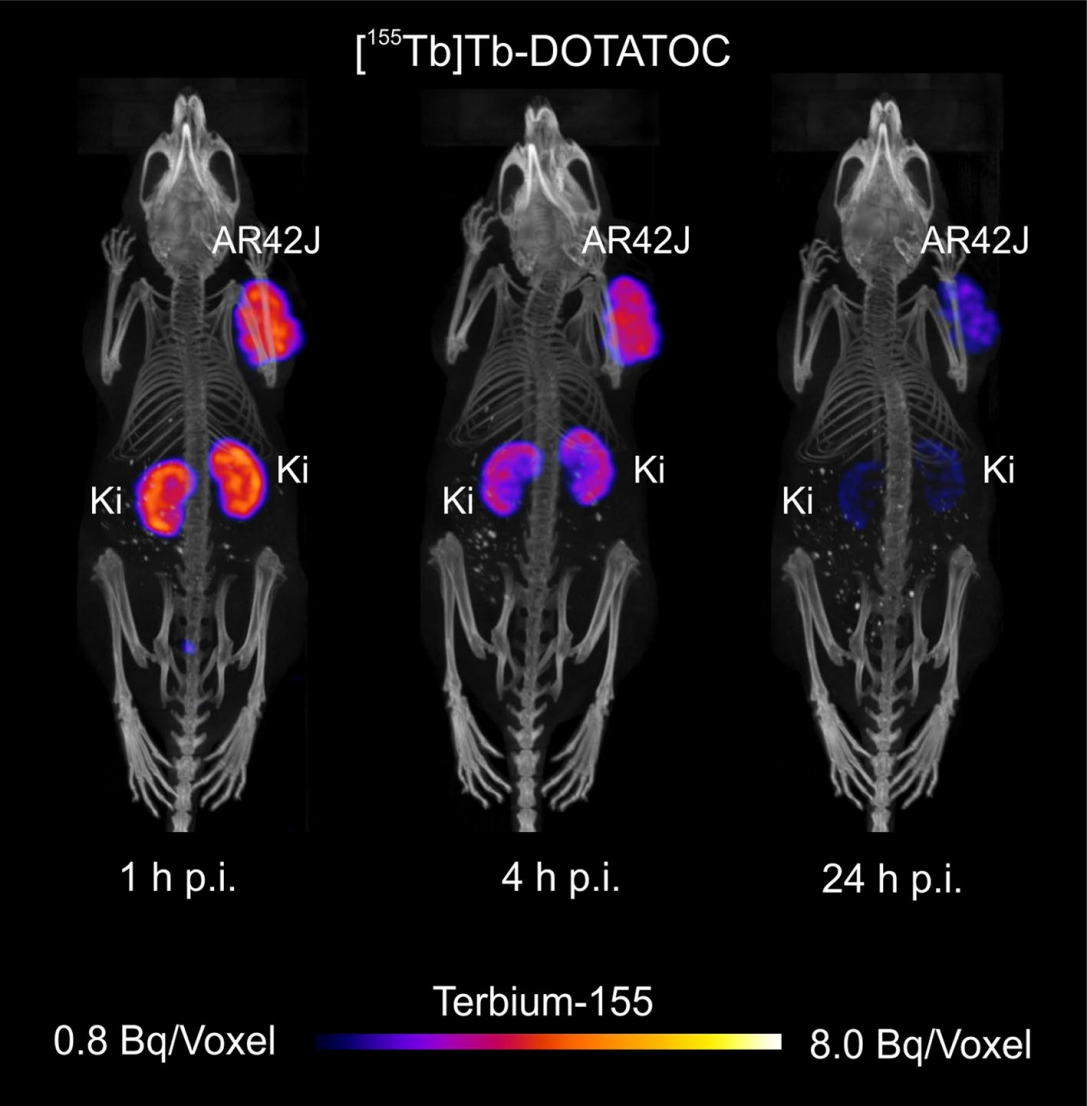
In vivo SPECT/CT scans of AR42J tumor-bearing mouse injected with [^155^Tb]Tb-DOTATOC (10 MBq, 1.0 nmol; 100 μL). The scans were acquired 1, 4 and 24 h pi. During the scans the mouse was anesthetized with a mixture of isoflurane and oxygen. (AR42J = SSTR-positive tumor xenograft, Ki = kidney). (Voxel size: 0.3 mm)

In vivo imaging using [^155^Tb]Tb-DOTATOC allowed excellent tumor visualization (Fig. 7). The ^155^Tb-radiolabeled SST analogue accumulated in AR42J tumor already after 1 h p.i. The activity in the tumor was still high at 4 h p.i., while it decreased after 24 h p.i.. The renal retention of [^155^Tb]Tb-DOTATOC was high shortly after injection, but the activity was cleared efficiently over time.

## Discussion

In this study, the first reproducible production of GBq activities of terbium-155 via the ^155^Gd(p,n)^155^Tb and ^156^Gd(p,2n)^155^Tb nuclear reactions, to our knowledge, was performed. In contrast to previous studies in which the production of terbium-155 (in MBq activities) was performed by spallation followed by mass separation (Fiaccabrino et al. 2021; Müller et al. 2012, 2014a; Webster et al. 2019), the current study was able to demonstrate the possibility of producing terbium-155 activities in view of clinical application. The production via ^155^Gd(p,n)^155^Tb was carried out both at a research cyclotron and a medical cyclotron, while the ^156^Gd(p,2n)^155^Tb was achieved at a research cyclotron only, due to the higher beam energy required (it must be noted that this production route is possible with commercial cyclotrons that can produce 24 MeV protons). The results obtained using a medical cyclotron represent a promising important step towards the implementation of terbium-155 production in quality and quantity suitable for application in nuclear medicine. However, as expected based on previously reported investigations, the ^156^Gd(p,2n)^155^Tb nuclear reaction generated higher terbium-155 activities than the ^155^Gd(p,n)^155^Tb nuclear reaction (. 2021a; Dmitriev et al. 1989; Vermeulen et al. 2012). A note of caution may be due here since the production yields may not be optimal: the IP2 research beamline requires degrading of the proton beam and this would result in beam spread (van der Meulen et al. 2019, 2020a); however, the majority of the production yields were reproducible.

Furthermore, after the purification process, it was possible to obtain up to 1.0 GBq of [^155^Tb]TbCl_3_ with a high level of chemical purity enabling labeling of biomolecules at up to 100 MBq/nmol. When the radiolabeling at high molar activities failed, a correlation with a low activity concentration was observed, implying that at low terbium-155 activities it was easy for metal environmental contaminants, mainly Zn and Fe, to compete with terbium-155 for the free chelator sites of DOTATOC (Asti et al. 2012; Talip et al. 2021, 2020). This hypothesis was also confirmed by means of LC–MS-ESI analysis of the radiolabeled samples. At higher terbium-155 activities, the analysis attested that the purification process of terbium-155 from the target material developed in this work was successful and effective. In particular, the purification process consisted of a three-column procedure that guaranteed the separation of a very small amount of terbium (1.7 GBq terbium-155 corresponds to ~ 1 µg of terbium) from a massive amount of gadolinium (40 mg of target material). The current method represents an improved process with respect to time as well as product purity, compared to that previously reported by our group (Gracheva et al. 2019), due to the introduction of a third column and further purification steps. The use of the DGA extraction resin column spared a time-consuming evaporation step before loading the reduced quantity of target material, along with the desired product, onto the Sykam resin column. In the elution profile of the DGA column, a partial separation between terbium and gadolinium was observed, resulting in a 50% reduction of gadolinium loaded onto the Sykam column, with a consequent higher separation efficiency and reproducibility. This implies the possibility of increasing the mass of target material to irradiate, which could allow for higher production yields. Another advantage of the revised method is that the final column, containing LN3 extraction resin, was used as a last purification step in where any remaining traces of gadolinium could be removed with 0.01 M HCl before final elution of terbium-155. The separation yield resulted in 75–90%, indicating that only 10–25% of terbium-155 activity were lost during separation, predominantly on the DGA resin column and final rinsing steps.

In contrast to the mass-separated terbium-155, the product in the present study (from both nuclear reactions) was obtained together with terbium-154, terbium-154m, terbium-154m2 and terbium-156. The radionuclidic purity was demonstrated to be dependent on the nuclear reaction used for terbium-155 production. In particular, when the ^156^Gd(p,2n)^155^Tb nuclear reaction was induced, the fraction of terbium-156 was measured as ~ 8% at EOS, which decreased to ~ 6% when the ^155^Gd(p,n)^155^Tb reaction was utilized. The longer-lived radionuclidic impurity terbium-156 has an almost identical half-life to the radioisotope of interest (Additional file 1: Table S5), making it impossible to be removed from the final product by decay. The 154 isobars are, however, short-lived radionuclidic impurities [T_1/2_ < 23 h (Reich 2009)], implying that the impurity could be eliminated after less than one week of decay. In future, the option of purifying the final product using an off-line mass separation technique could be evaluated (Talip et al. 2021) to separate terbium-156 from terbium-155, in order to increase the radionuclidic purity, even though this approach would inevitably reduce the final production yields due to the currently poor efficiency of ionization. To develop a full picture of the topic, additional studies will be needed to include the dosimetry of terbium-156 in a hypothetical clinical setting, in order to establish the right compromise between production outcome and radiological safety.

Furthermore, in this work, a Derenzo Phantom SPECT study was performed with terbium-155, which was comparable to that previously obtained with the mass-separated terbium-155 and approximately same collected counts (Müller et al. 2014a), meaning that the presence of other terbium radioisotopes appeared not to affect the visual resolution of the picture. Moreover, preliminary cell experiments confirmed that the behavior of [^155^Tb]Tb-DOTATOC was comparable to that of the previously reported for its ^177^Lu-radiolabeled counterpart (Additional file 1: Fig S10) (Borgna et al. 2021), and allowed further in vivo studies. Importantly, the SPECT/CT in vivo imaging study, using the mentioned radiolabeled SST analogue, demonstrated the applicability of cyclotron-produced terbium-155 for imaging purposes along with its excellent feature for visualization of the accumulated radiolabeled compound in the tumors. In particular, the in vivo distribution was comparable with that of the same ^177^Lu- or ^161^ Tb-labeled biomolecule previously performed (Borgna et al. 2021). As a result, terbium-155 could play an important role towards the application of radiotheragnostics in the field.

## Conclusions

In the present study, it was demonstrated that high activities of terbium-155 can be produced via proton irradiation of highly enriched gadolinium-155 or gadolinium-156 oxide targets. The ^156^Gd(p,2n)^155^Tb nuclear reaction demonstrated higher production yields, however, lower radionuclidic purity in comparison with the final product of the ^155^Gd(p,n)^155^Tb nuclear reaction was observed. As the latter method would be feasible on a medical cyclotron and, thus, more likely to be used in future, it remains to be determined as to how these co-produced terbium radioisotopes can affect patients from a dosimetry perspective. The high chemical purity of the final separated product ([^155^Tb]TbCl_3_), ensured radiolabeling of tumor targeting molecules, which would be necessary for clinical application. The results of this research support the idea that cyclotron-produced terbium-155 is a promising radionuclide for medical purposes and lay the groundwork for further preclinical in vitro and in vivo investigations, together with future potential clinical applications.

## Supplementary Information

**Additional file 1**. Supplementary Material.

## Data Availability

The dataset(s) supporting the conclusions of this article is (are) included within the article (and its additional file(s)).

## Abbreviations

SPECT: Single photon emission computed tomography
EOB: End of bombardment
BTL: Beam transfer line
STS: Solid target station
STTS: Solid target transfer system
CZT: CdZnTe crystal
LHEP: Laboratory of high energy physics
EOS: End of separation
DGA: N,N,N′,N′-tetra-n-octyldiglycolamide extraction resin
LN3: Bis(2,4,4-trimethyl-1-pentyl) phosphinic acid extraction resin
α-HIBA: α-Hydroxy-isobutyric acid
HPGe: High-purity Germanium
HPLC: High performance liquid chromatography
LC-MS-ESI: Liquid chromatography-mass spectrometry-electrospray ionization
DTPA: Diethylenetriamine pentaacetic acid
SST: Somatostatin
