# Supplementary material for: Cyclotron production and radiochemical purification of terbium-155 for SPECT imaging
Source: EJNMMI Radiopharm Chem. 2021 Nov 14;6:37. doi: 10.1186/s41181-021-00153-w (PMC8590989; doi:10.1186/s41181-021-00153-w)
Supplement: Supplementary file 1 — Additional file 1. Supplementary Material. [file 41181_2021_153_MOESM1_ESM.docx]

Additional file 1

**Cyclotron production and radiochemical purification of terbium-155 for SPECT imaging**

C. Favaretto^1,4^, Z. Talip^1^, F. Borgna^1^, P. V. Grundler^1^, G. Dellepiane^2^, H. Zhang^3^, A. Sommerhalder^1^, R. Schibli^1,4^, S. Braccini^2^, C. Müller^1^, N. P. van der Meulen^1,5*^

^1^Center for Radiopharmaceutical Sciences ETH-PSI-USZ, Paul Scherrer Institut, 5232 Villigen-PSI, Switzerland

^2^ Albert Einstein Center for Fundamental Physics (AEC), Laboratory of High Energy Physics (LHEP), University of Bern, 3012 Bern, Switzerland

^3^Division Large Research Facilities, Paul Scherrer Institut, 5232 Villigen-PSI, Switzerland

^4^Department of Chemistry and Applied Biosciences, ETH Zurich, 8093 Zurich, Switzerland

^5^Laboratory of Radiochemistry, Paul Scherrer Institut, 5232 Villigen-PSI, Switzerland

E-mail addresses:

***Corresponding author**:

Dr. Nicholas P. van der Meulen

Laboratory of Radiochemistry/Center for Radiopharmaceutical Sciences ETH/PSI/USZ

Paul Scherrer Institut

5232 Villigen-PSI

Switzerland

e-mail: nick.vandermeulen@psi.ch

phone: +41-56-310 50 87

fax: +41-56-310 28 49

1. **Enrichment level and isotopic distribution of target material**

The enrichment levels and the full isotopic distribution of the target material used in this study ([^155^Gd]Gd_2_O_3_ and [^156^Gd]Gd_2_O_3_) were provided by the manufacturer (Isoflex, USA) and are summarized in Tables S1 and S2.

**Table S1** Enrichment levels and isotopic distribution obtained from the Certificate of Analysis of [^155^Gd]Gd_2_O_3_.

| **Isotope** | [^152^Gd]Gd | [^154^Gd]Gd | [^155^Gd]Gd | [^156^Gd]Gd | [^157^Gd]Gd | [^158^Gd]Gd | [^160^Gd]Gd |
| --- | --- | --- | --- | --- | --- | --- | --- |
| **Content (%)** | <0.02 | 0.5 | 91.90 | 5.87 | 0.81 | 0.65 | 0.27 |

**Table S2** Enrichment levels and isotopic distribution obtained from the Certificate of Analysis of [^156^Gd]Gd_2_O_3_.

| **Isotope** | [^152^Gd]Gd | [^154^Gd]Gd | [^155^Gd]Gd | [^156^Gd]Gd | [^157^Gd]Gd | [^158^Gd]Gd | [^160^Gd]Gd |
| --- | --- | --- | --- | --- | --- | --- | --- |
| **Content (%)** | <0.01 | 0.5 | 0.87 | 93.30 | 4.38 | 1.08 | 0.32 |

1. **Terbium-155 production**

*Purpose:* In this section, the complete dataset of terbium-155 productions performed both at the PSI’s Injector 2 cyclotron and at the Bern medical cyclotron are reported.

*Method:* Three [^155^Gd]Gd_2_O_3_ targets and six [^156^Gd]Gd_2_O_3_ targets (prepared as described in the main manuscript) were irradiated, using the 72 MeV proton beam from the Injector 2 separated sector cyclotron. The beam current was set to 50 µA and niobium discs (3.4 mm and 2.4 mm, respectively) were used as degraders to decrease the proton energy from 72 MeV to ~10.3 MeV for the ^155^Gd(p,n)^155^Tb reaction and ~22.8 MeV for the ^156^Gd(p,2n)^155^Tb. The irradiations were performed for 1, 2, 4 and 8 hours.

Two additional irradiations were performed at the Bern medical cyclotron (IBA Cyclone HC 18 MeV) in operation at the Bern University Hospital (Inselspital) on [^155^Gd]Gd_2_O_3_ targets. The irradiations were performed at 2.4 µA and 0.7 µA beam intensity with 10.9 MeV and 10.5 MeV proton energy, respectively. The entry and exit energies were tuned by adjusting the thickness of the covering lid of the coin. The irradiations were conducted for 70 and 40 minutes, respectively.

*Results:* The terbium-155 activity produced for each production are reported in Tables S3 and S4 with the correspondent parameters of irradiation. The most obvious finding to emerge from the analysis is that the ^156^Gd(p,2n)^155^Tb nuclear reaction yielded higher activities compared to the ^155^Gd(p,n)^155^Tb reaction. In addition, the terbium-155 production obtained using a medical cyclotron represents a promising step towards the implementation of terbium-155 production in quantity suitable for potential nuclear medicine application in a clinical environment.

**Table S3** Terbium-155 activities produced via ^155^Gd(p,n)^155^Tb and ^156^Gd(p,2n)^155^Tb nuclear reactions at PSI’s Injector 2 cyclotron. Beam entry and exit energies were calculated using SRIM.

| Production No | Nuclear reaction | Target mass [mg] | Current on pellet [µA] | Beam entry energy [MeV] | Beam exit energy [MeV] | Irradiation time [h] | ^155^Tb Activity EOB [MBq] |
| --- | --- | --- | --- | --- | --- | --- | --- |
| 1 | ^155^Gd(p,n)^155^Tb | 41 | 50 | 10.3 ± 2.6 | 6.0 ± 3.4 | 1 | 31.4 |
| 2 | ^155^Gd(p,n)^155^Tb | 39 | 50 | 10.3 ± 2.6 | 6.0 ± 3.4 | 4 | 26.8 |
| 3 | ^155^Gd(p,n)^155^Tb | 39 | 50 | 10.3 ± 2.6 | 6.0 ± 3.4 | 8 | 202 |
| 4 | ^156^Gd(p,2n)^155^Tb | 39 | 50 | 22.8 ± 1.3 | 20.7 ± 1.5 | 2 | 306 |
| 5 | ^156^Gd(p,2n)^155^Tb | 40 | 50 | 22.8 ± 1.3 | 20.7 ± 1.5 | 4 | 705 |
| 6 | ^156^Gd(p,2n)^155^Tb | 41 | 50 | 22.8 ± 1.3 | 20.7 ± 1.5 | 4 | 705 |
| 7 | ^156^Gd(p,2n)^155^Tb | 39 | 50 | 22.8 ± 1.3 | 20.7 ± 1.5 | 8 | 1250 |
| 8 | ^156^Gd(p,2n)^155^Tb | 39 | 50 | 22.8 ± 1.3 | 20.7 ± 1.5 | 8 | 1684 |
| 9 | ^156^Gd(p,2n)^155^Tb | 39 | 50 | 22.8 ± 1.3 | 20.7 ± 1.5 | 8 | 900 |

**Table S4** Terbium-155 activities produced via ^155^Gd(p,n)^155^Tb at the Bern medical cyclotron. Beam entry and exit energies were calculated using SRIM.

| Production No | Nuclear reaction | Target mass [mg] | Current on pellet [µA] | Beam entry energy [MeV] | Beam exit energy [MeV] | Irradiation time  [min] | ^155^Tb Activity EOB [MBq] |
| --- | --- | --- | --- | --- | --- | --- | --- |
| 1 | ^155^Gd(p,n)^155^Tb | 38 | 2.4 ± 0.7 | 10.9 ± 0.4 | 7.8 ± 0.6 | 70 | 7.7 ± 0.6 |
| 2 | ^155^Gd(p,n)^155^Tb | 38 | 0.7 ± 0.2 | 10.5 ± 0.4 | 7.1 ± 0.7 | 40 | 1.6 ± 0.1 |

1. **Development of terbium-155 purification process on the bench**

**Evaluation of terbium and gadolinium elution profiles from DGA and Sykam resins**

*Purpose:* After the irradiation of the gadolinium oxide targets, a chemical separation of the terbium-155 produced from its target material is necessary. The process was accurately established with several bench experiments. In particular, the elution profiles of terbium and gadolinium were evaluated with long-lived radioactive tracers and stable isotopes of the two lanthanides in question on two ion exchange resins (i.e. DGA extraction resin and Sykam cation exchange resin).

*Method:* Radioactive tracers of terbium-160 and gadolinium-153 (produced via neutron irradiation at SINQ, Paul Scherrer Institute (PSI), Switzerland) were mixed together with 5 µg of terbium (5 µL of 1000 ppm Sigma Aldrich tracer for ICP) and 40 mg of natural Gd_2_O_3_ (Research chemicals, Division of Rhone-Poulenc Inc., USA) in 5.0 mL 7.0 M HNO_3_ (Suprapur, Merck, Germany). An irradiated target dissolution was simulated with the solution described and then loaded onto the DGA resin column (DGA Normal resin, Triskem International, France; particle size 50–100 μm, column volume: 8.5 mL) using an Ismatec peristaltic pump (Cole-Parmer Instrument Company LLC, USA). After loading the resin, terbium and gadolinium were eluted with 45-50 mL 0.05 M HCl (Suprapur, Merck, Germany) collected in 3 mL fractions. The fractions were then measured by γ-ray spectrometry using a high-purity germanium (HPGe) detector (Canberra, France) in combination with the InterWinner software package (version 7.1, Itech Instruments, France). Furthermore, the fractions containing terbium-160 were collected together and then loaded onto a Sykam resin column (Sykam Chromatographie Vertriebs GmbH, Germany; particle size 12–22 μm, NH_4_^+^ form, column volume: 19.8 mL). Subsequently, 0.13 M (pH 4.5) α-hydroxy-isobutyric acid (α-HIBA, Sigma-Aldrich GmbH, Germany) was pumped through the column until terbium was eluted. The concentration of α-HIBA was then increased to 1.0 M to elute gadolinium. Samples (3 mL fractions) of α-HIBA were collected and measured using the HPGe detector until the 3σ uncertainty was below 10%. The same experiment was conducted exclusively using stable isotopes. Therefore, 5 µg of terbium and 40 mg of natural Gd_2_O_3_ were mixed and loaded to both columns, as described above, without the addition of radiotracers. The concentration of the lanthanides in the 5 mL fractions taken from the DGA and Sykam eluents, respectively, was measured by means of ICP-OES (Agilent 5110 ICP-OES, USA). Both sets of experiments were performed at least 3 times.

*Results:* With the data collected in these experiments, the elution profiles and separation factors of terbium and gadolinium from DGA and Sykam resins were defined. In particular, according to the measured counts of radioactive tracers in the collected fractions, the elution profile of the two lanthanides under the described conditions was established (Fig. S1). Based on the indication given by the tracer experiments, further experiments with stable isotopes were conducted and, from the subsequent ICP-OES measurements of the fractions collected, it was possible to obtain a more accurate measure of terbium and gadolinium in all the fractions and more precise elution profiles from DGA and Sykam columns under the specific experimental conditions (Fig. S2). Moreover, with the data obtained from the ICP-OES measurements it was possible to calculate the gadolinium/terbium separation factor for the DGA separation step, which resulted 2.00 ± 0.07.


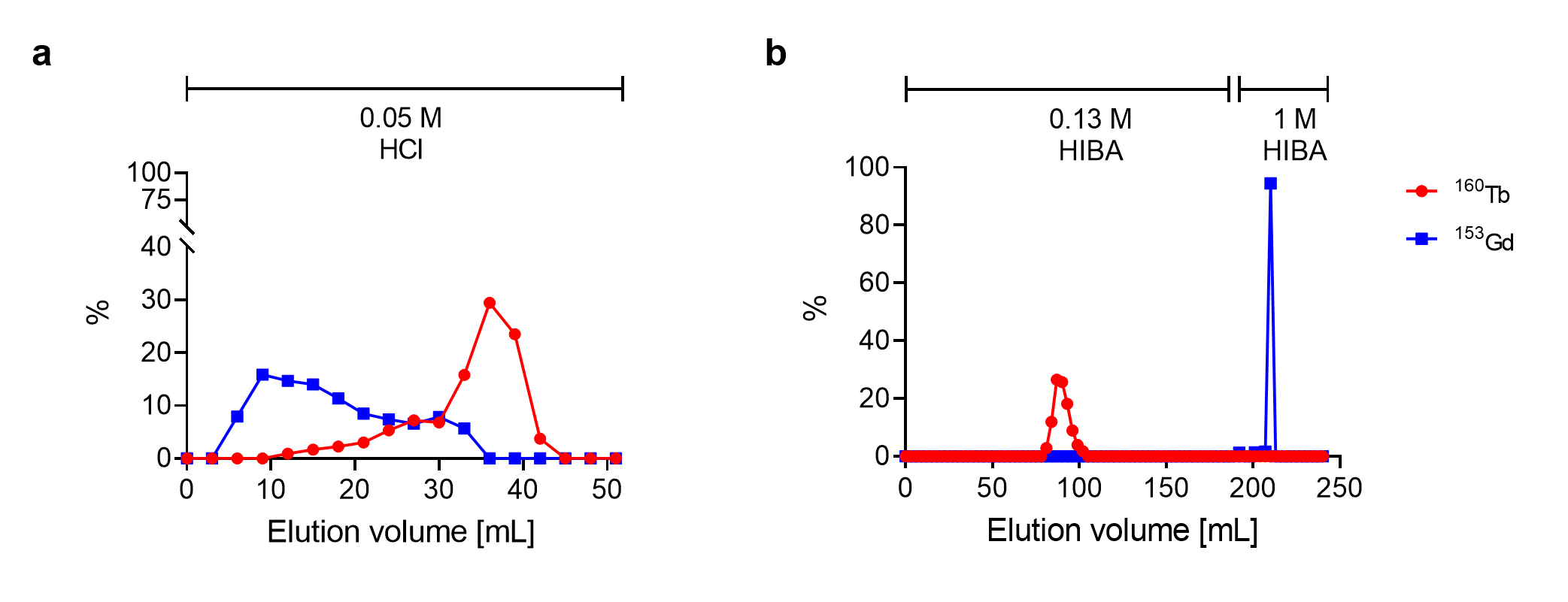


**Fig. S1** Elution profile of terbium-160 and gadolinium-153 from DGA (**a**) and Sykam resin (**b**) (separation profiles were obtained based on gamma-ray spectrometry measurements of the eluted volumes). The experiment was performed with the addition of 5 µg terbium and 40 mg ^nat^Gd_2_O_3_ as carriers to simulate the real conditions of terbium-155 purification after target irradiation.


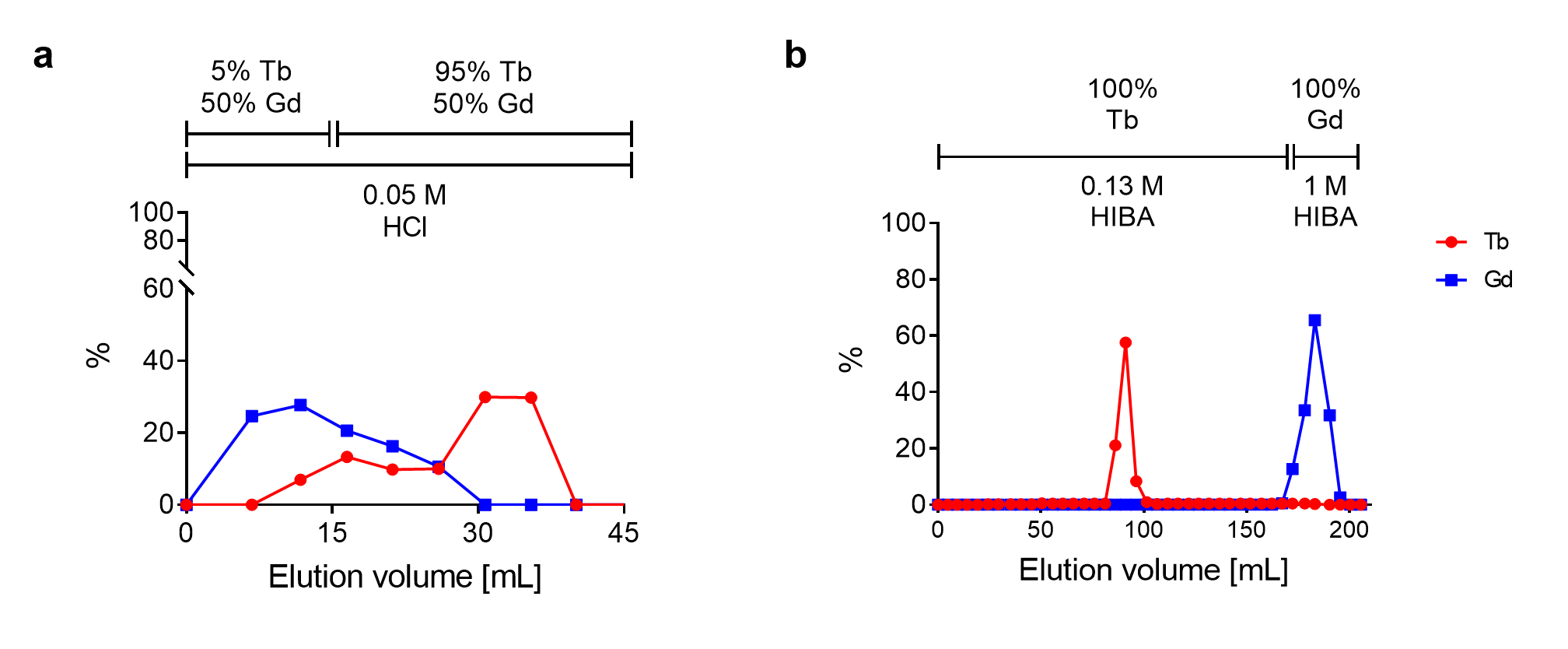


**Fig. S2** Elution profile of 5 µg of terbium and 40 mg of ^nat^Gd_2_O_3_ from DGA (**a**) and Sykam resin (**b**) (separation profiles were obtained based on ICP-OES measurements of the eluted volumes).

**Evaluation of terbium and gadolinium elution profiles from Sykam resin with increasing amounts of gadolinium**

*Purpose***:** The variation of the elution profiles of terbium and gadolinium from the Sykam cation exchange resin column was evaluated with long-lived radioactive tracers and increasing amounts of gadolinium oxide.

*Method:* Radioactive tracers of terbium-160 and gadolinium-153 were mixed together with 5 µg of terbium and 5 µg, 50 mg or 100 mg of natural Gd_2_O_3_ in 5.0 mL 0.05 M HCl, to simulate the solution obtained from the dissolution of irradiated targets of variable sizes. The solution was then loaded onto a 10 mL Sykam resin column by means of a peristaltic pump. Eventually, terbium and gadolinium were eluted with 0.11 M α-HIBA (pH 4.5) until terbium was completely removed from the column, followed by 0.14 M α-HIBA (pH 4.5) until gadolinium was eluted too. The eluted volume was collected in 3 mL fractions, which were measured using the HPGe detector one after the other during the elution process.

*Results*: Thanks to the counts measured in the fractions collected during this experiment, the elution profiles of terbium and gadolinium with variable amounts of gadolinium were defined (Fig. S3). The clearest finding to derive from the analysis is that increasing amounts of gadolinium oxide (target material) produce less resolute elution peaks for both of the lanthanides, drastically reducing the separation efficiency between them.


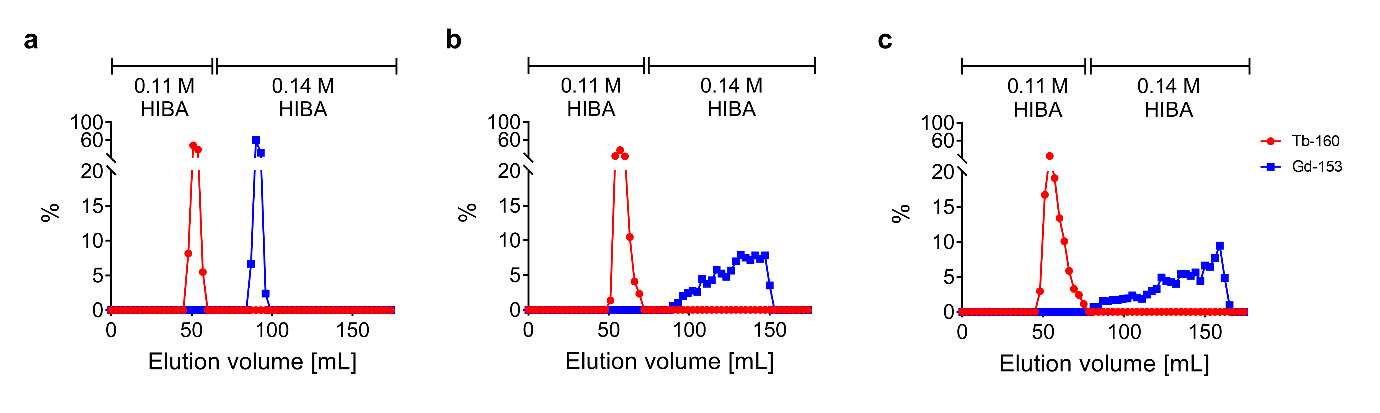


**Fig. S3** Separation of 5 µg of terbium from 5 µg (**a**), 50 mg (**b**) and 100 mg (**c**) of ^nat^Gd_2_O_3_ using Sykam resin and α-HIBA as eluent (separation profiles were obtained based on γ-spectrometry measurements of the lanthanide radiotracers contained in the eluted volumes).

**Evaluation of terbium and gadolinium elution profiles from LN3 resin**

*Purpose:* In order to ensure a reproducible terbium-155 purification process, the last purification step of the method, performed with bis(2,4,4-trimethyl-1-pentyl)phosphinic acid extraction resin (LN3, Triskem International, France; 6mm× 5 mm), was thoroughly investigated. In particular, the elution profile and the possible terbium-gadolinium separation using LN3 resin column were evaluated with different eluents and flow rates.

*Method:* Long-lived radioactive tracers of terbium-160 and gadolinium-153 were mixed together in 25 mL 0.13 M α-HIBA (pH 4.5) and loaded onto a ~ 0.08 mL LN3 resin column. With this procedure, the solution eluted from the Sykam column and loaded onto the LN3 column of the terbium-155 purification method was simulated. Terbium-160 and gadolinium-153 were then eluted from LN3 with 1 mL 0.05 M HCl at 0.1 mL/min according to the procedure used by Gracheva et al. (Gracheva et al. 2019), and collected in 0.1 mL fractions. In a further experiment, after the loading of an LN3 column of the same size, the remaining gadolinium was eluted with 19 mL 0.01 M HCl, followed by 1 mL 0.05 M HCl. The eluents were eluted at 0.1 mL/min and collected in 0.5 mL fractions. The radioactive tracers in all the fractions were measured by γ-ray spectrometry using the HPGe detector previously mentioned.

Furthermore, the influence of the flow rate on the elution of terbium and gadolinium from LN3 resin was assessed with the use of stable isotopes and ICP-OES measurements. Two solutions containing 5 µg of terbium and 5 µg of gadolinium in 25 mL 0.13 M α-HIBA (pH 4.5) were loaded onto two LN3 columns, respectively. To one column, 14 mL 0.01 M HCl was applied at a flow rate of 0.1 mL/min, while in the other one the same volume of solution was applied at 1 mL/min. After this step, both of the columns were eluted with 1 mL 0.05 M HCl at 0.1 mL/min. The eluents were collected in 1 mL fractions and the lanthanides’ content analyzed by means of ICP-OES.

*Results*: With the first series of experiments it was demonstrated that the last step of the purification process, performed with LN3 extraction resin, could be used not only to concentrate the final product but also as a last purification step between terbium and gadolinium, in order to ensure the removal of all the gadolinium tracers and the reproducibility of the terbium-155 product chemical purity. In particular, when a rinse step with 0.01 M HCl was performed before the final terbium-155 elution, traces of gadolinium, potentially co-eluted with terbium from the Sykam column, could be efficiently separated from the final product (Fig. S4b). This outcome is clearly an improvement of the previously-reported method (Gracheva et al. 2019), where gadolinium was potentially co-eluted with terbium from the LN3 column in 1 mL 0.05 M HCl (Fig. S4a).


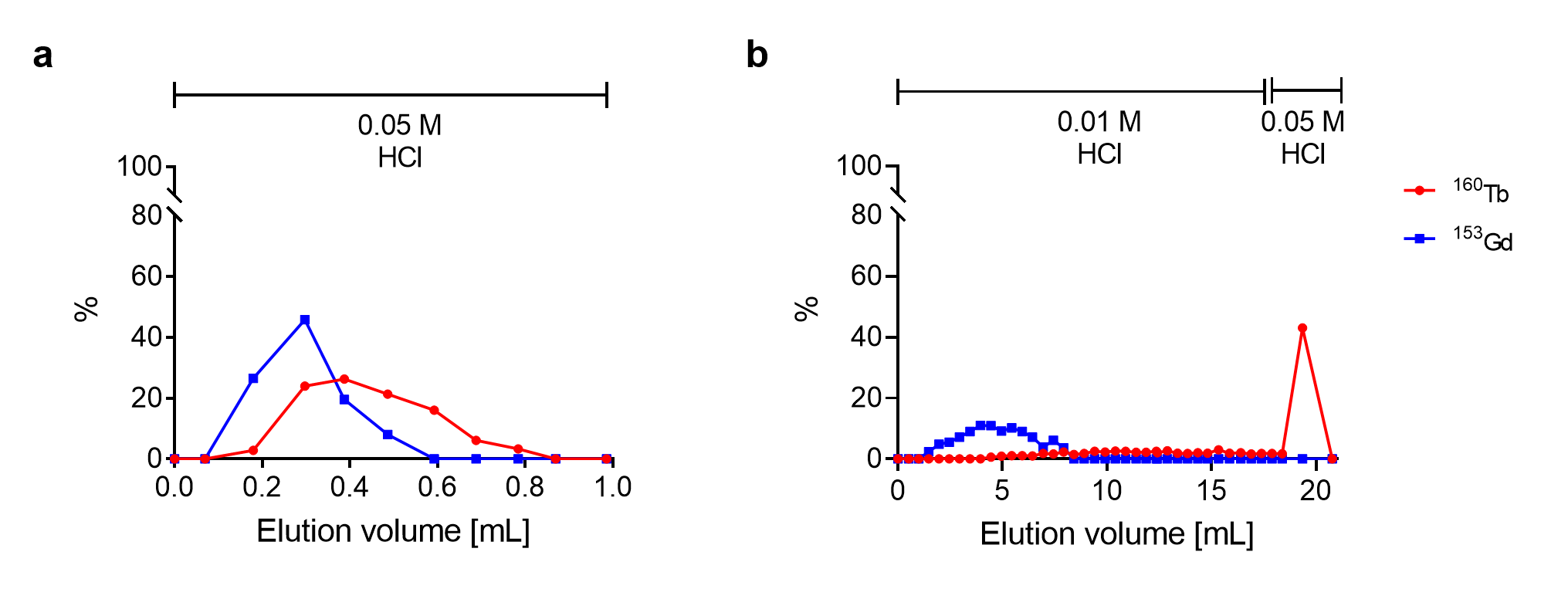


**Fig. S4** Elution profiles and separation between terbium-160 and gadolinium-153 using LN3 resin and 0.05 M HCl (**a**) or 0.01 M followed by 0.05 M HCl (**b**) (separation profiles were obtained based on γ-spectrometry measurements of the eluted fractions).

The flow rate was demonstrated to have an influence on the elution profiles of terbium and gadolinium from the LN3 column. In particular, when 0.01 M HCl was eluted at 0.1 mL/min, ~80% of gadolinium was removed from the column, resulting in a gadolinium/terbium separation factor of 4.5 (Fig. S5a), while when the same solution was eluted at 1 mL/min the gadolinium removed from the column was ~15% less (separation factor 2.6) (Fig. S5b). When working with radioactive material, it is always important to consider the time consumption factor which, in the case of the lower flow rate, would be negatively impacted. Moreover, since it is believed that gadolinium should only be in tracer quantities (at worst) at this stage, 65% decrement of gadolinium is sufficient to ensure a final product (^155^TbCl_3_) chemically pure enough to guarantee efficient radiolabeling of biomolecules (Asti et al. 2012).

As a result of this investigation, the use of a 14-mL 0.01 M HCl rinse step at 1 mL/min was introduced to the established purification process before the final elution of terbium-155.


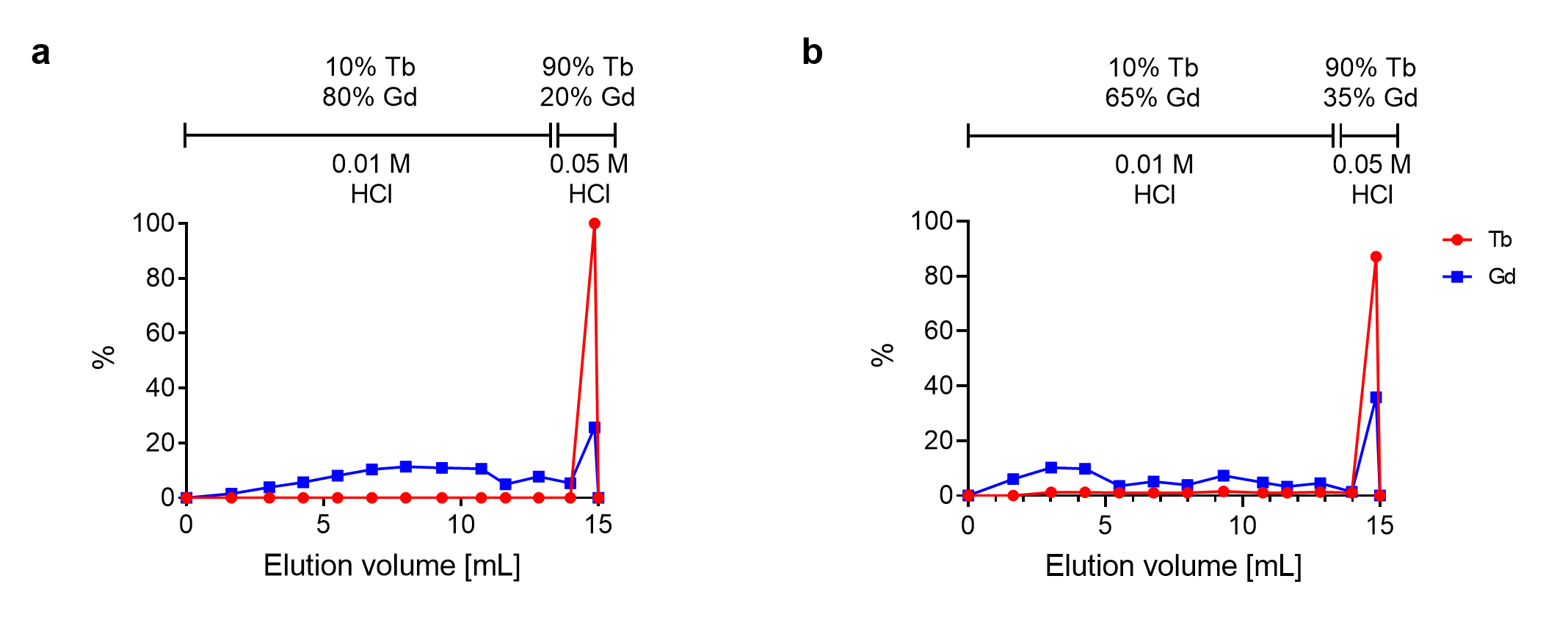


1. **Fig. S5** Elution profiles and separation between terbium and gadolinium using LN3 resin and 0.01 M HCl 0.1 mL/min (**a**) or 0.01 M HCl at 1 mL/min (**b**) before 0.05 M HCl at 0.1 mL/min (separation profiles were obtained based on ICP-OES measurements of the eluted volumes).**Evaluation of terbium-155 radionuclidic purity**

*Purpose:* The aim of this experiment was to assess the radionuclidic purity of terbium-155 produced via ^155^Gd(p,n)^155^Tb and ^156^Gd(p,2n)^155^Tb nuclear reactions through γ-ray spectrometry measurements.

*Method:* The terbium-155 activities were determined before and after separation by γ-ray spectrometry using a high-purity germanium (HPGe) detector (Canberra, France), in combination with the Inter-Winner software package (version 7.1, Itech Instruments, France). The efficiency calibration was performed using an Eppendorf vial filled with a europium-152 solution (89.51 kBq ± 0.71%, reference date 20.02.2017) and placed at 1 m from the detector. A 5 µL aliquot of the final product (^155^TbCl_3_) was introduced in an Eppendorf vial and measured with uncertainty ≤5% at 1 m from the detector.

*Results*: The γ-ray spectrum obtained showed the γ-lines of terbium-155 together with those of terbium-156, terbium-154, terbium-154m and terbium-154m2 (Table S5-S6 and Fig. S6).

**Table S5** The γ-lines of terbium-155 and its radioisotopes terbium-156, terbium-154, terbium-154m and terbium-154m2 (Nica 2019; Reich 2012; Reich 2009).

| Radioisotope | Terbium-155 | Terbium-156 | Terbium-154 | Terbium-154m | Terbium-154m2 |
| --- | --- | --- | --- | --- | --- |
| Half-life | 5.32 d | 5.35 d | 21.5 h | 9.4 h | 22.7 h |
| Eγ [keV] (Iγ) | 86.55 (32%)  105.318 (25.1%)  180.08 (7.5%)  262.27 (5.3%) | 534.29 (67%)  199.19 (41%)  1222.44 (31%)  88.97 (18%)  356.38 (13.6%)  1421.67 (12.2%)  1065.11 (10.8%)  1154.07 (10.4%)  422.34 (8%)  1159.03 (7.2%)  262.54 (5.8%) | 123.07 (26%)  1274.436 (10.5%)  2187.10 (9.9%)  722.12 (7.7%)  1996.61 (7.5%)  2061.11 (7.1%)  1291.31 (6.9%)  1123.09 (5.7%)  557.60 (5.4%)  873.21 (5.3%) | 123.07 (30%)  247.93 (22.1%)  540.18 (20%)  649.56 (10.9%)  1004.73 (10.9%)  873.19 (9.2%)  996.26 (8.6%)  518.01 (6.1%) | 247.93 (79%)  346.64 (69%)  1419.81 (46%)  123.07 (43%)  225.94 (26.8%)  426.78 (17%)  992.92 (16.2%)  649.56 (8.6%) |

Only the lines with Iγ > 5% are reported.

**Table S6** Activities of terbium-155 and radionuclidic impurities after purification of selected targets (~2 days after EOB). Production No 3 and 6 were excluded due to technical failures of the separation panel.

| Production No | Nuclear reaction | Irradiation time [h] | [^155^Tb]Tb EOS [MBq] | [^156^Tb]Tb EOS [MBq] | [^154^Tb]Tb EOS [MBq] | [^154m^Tb]Tb EOS  [MBq] | [^154m2^Tb]Tb EOS  [MBq] |
| --- | --- | --- | --- | --- | --- | --- | --- |
| 2 | ^155^Gd(p,n)^155^Tb | 4 | 18.1 | 1.25 | —* | —* | 0.0133 |
| 5 | ^156^Gd(p,2n)^155^Tb | 4 | 487 | 42.1 | 19.4 | —* | 2.14 |
| 7 | ^156^Gd(p,2n)^155^Tb | 8 | 829 | 71.3 | 25.1 | 19.0 | 2.68 |
| 8 | ^156^Gd(p,2n)^155^Tb | 8 | 1021 | 83.9 | 38.7 | 36.8 | 4.26 |
| 9 | ^156^Gd(p,2n)^155^Tb | 8 | 668 | 54.7 | 26.4 | 27.6 | 2.79 |

*Values were excluded due to the (too) high uncertainty of the measurement.


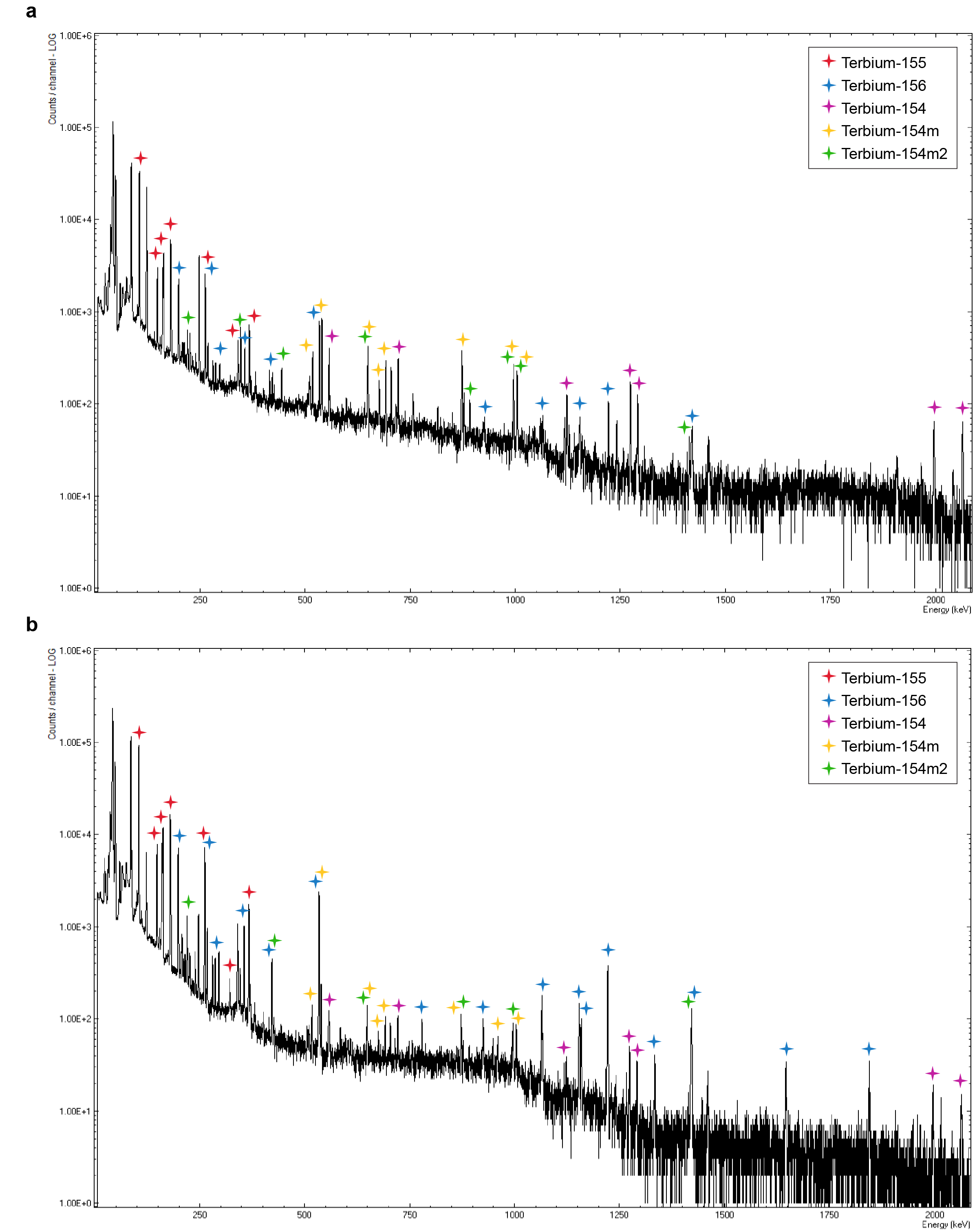


**Fig. S6** The γ-ray spectrum of ^155^TbCl_3_ solution obtained after the purification process of terbium-155 produced via the ^155^Gd(p,n)^155^Tb nuclear reaction (**a**) and ^156^Gd(p,2n)^155^Tb nuclear reaction (**b**). A 5 µL aliquot of the solution was measured EOS (~2 days after EOB) for 2 h in an Eppendorf vial at 1 m from the detector. Red star = terbium-155 peaks, blue star = terbium-156 peaks, violet star = terbium-154 peaks, yellow star = terbium-154m peaks, green star = terbium-154m2 peaks.

1. **Evaluation of terbium-155 chemical purity**

*Purpose:* After the production and purification of terbium-155, the chemical purity of the radionuclide was established assessing the radiolabeling yield with DOTATOC at high molar activities. Moreover, the radiolabeled samples, in the case of radiolabeling yields < 95%, were analyzed through liquid chromatography-mass spectrometry-electrospray ionization (LC-MS-ESI, Waters LCT Premier mass spectrometer) to demonstrate the partial radiolabeling of DOTATOC with terbium-155 and to identify the other metals labeled to the peptide.

*Methods:* For every separation process, the terbium-155 produced was used to radiolabel DOTATOC following a procedure previously described (Müller et al. 2014). Quality control was performed to determine the radiolabeled fractions of DOTATOC as previously reported by means of high-performance liquid chromatography (HPLC, Merck Hitachi, LaChrom) with a C-18 reversed-phase column (XterraTM MS, C18, 5 μm, 150 × 4.6 mm; Waters) and a radiodetector (Berthold, HPLC Radioactivity Monitor, LB 506B). The mobile phase consisted of MilliQ water containing 0.1% trifluoroacetic acid (A) and acetonitrile (B) with a gradient of 95% A and 5% B to 20% A and 80% B, for 15 min, at a flow rate of 1.0 mL/min. The sample for the analysis consisted of ~ 0.1 MBq of the radiolabeling solution in 1 mL MilliQ water containing sodium diethylenetriamine pentaacetic acid (Na-DTPA, 50 μM). For the estimation of the radiolabeling yields of [^155^Tb]Tb-DOTATOC from the obtained HPLC chromatogram, the product peak was integrated over the sum of all radioactive peaks (the radiolabeled product, potentially released activity subsequently bound to DTPA, as well as degradation products of unknown structure), which were set to 100% (Gracheva et al. 2019).

The radiolabeled samples which showed radiolabeling yield <95% were further analyzed by means of liquid chromatography-mass spectrometry (LC-MS-ESI, Waters LCT Premier mass spectrometer). The analyzed sample consisted of ~0.3 nmol of the radiolabeled peptide mixed with diethylenetriamine pentaacetic acid (Na-DTPA, 50 μM) for a total volume of 80 µL. The samples were chromatographed on a ReproSil-Pur 120 C18-AQ column (3 μm, 150 mm x 2 mm; Dr. Maisch GmbH, Germany). The following gradient was applied: 0-3 min with 2% A and 98% B; 3-17 min with 60% A and 40% B; 17-20 min with 85% A and 15% B (where A = acetonitrile containing 0.1% formic acid and B = water containing 0.1% formic acid) using a constant flow of 0.5 mL/min. MassLynx V4.1 was applied for data collection and MaxEnt1 for data deconvolution.

*Results:* Depending on the activity concentration of the ^155^TbCl_3_ solution, it was also feasible to label, with a radiolabeling yield >99%, at molar activities of up to 100 MBq/nmol (Fig. S7, Table 5).


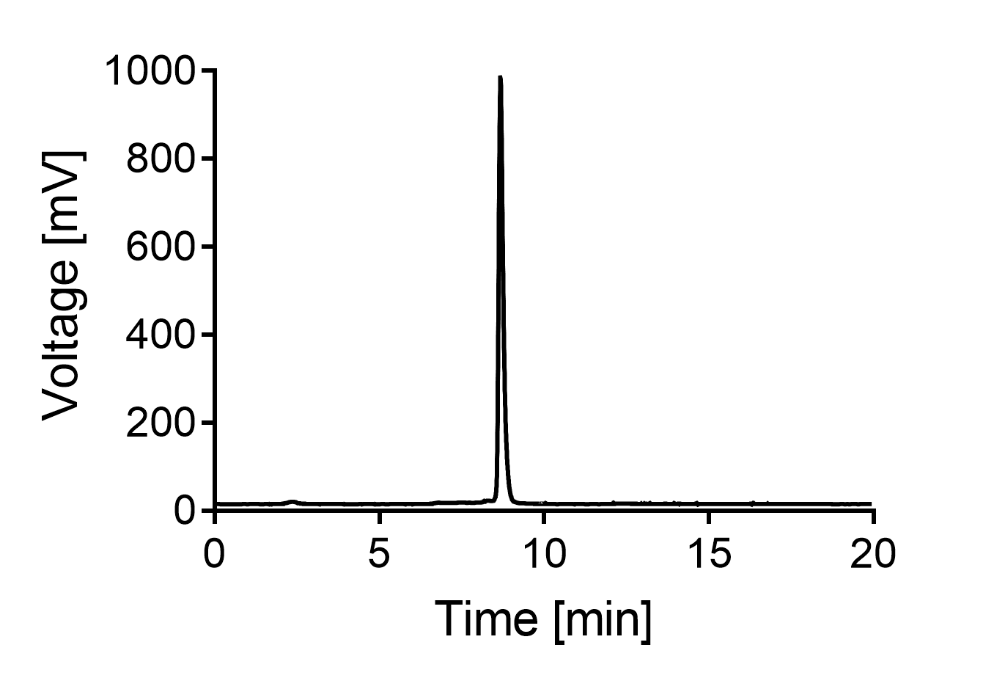


**Fig. S7** Radiodetector signal of a representative HPLC chromatogram of 100 MBq/nmol [^155^Tb]Tb-DOTATOC (2.3 min retention time would indicate “free” or unlabeled terbium-155, while 8.2 min indicates [^155^Tb]Tb-DOTATOC).

The LC-MS analysis of the radiolabeled samples with radiolabeling yield <95% didn’t show the clear presence of Gd-DOTATOC or [^155^Tb]Tb-DOTATOC, but it did prove the presence of Zn-DOTATOC and Fe-DOTATOC when poorly radiolabeled (Fig. S8).


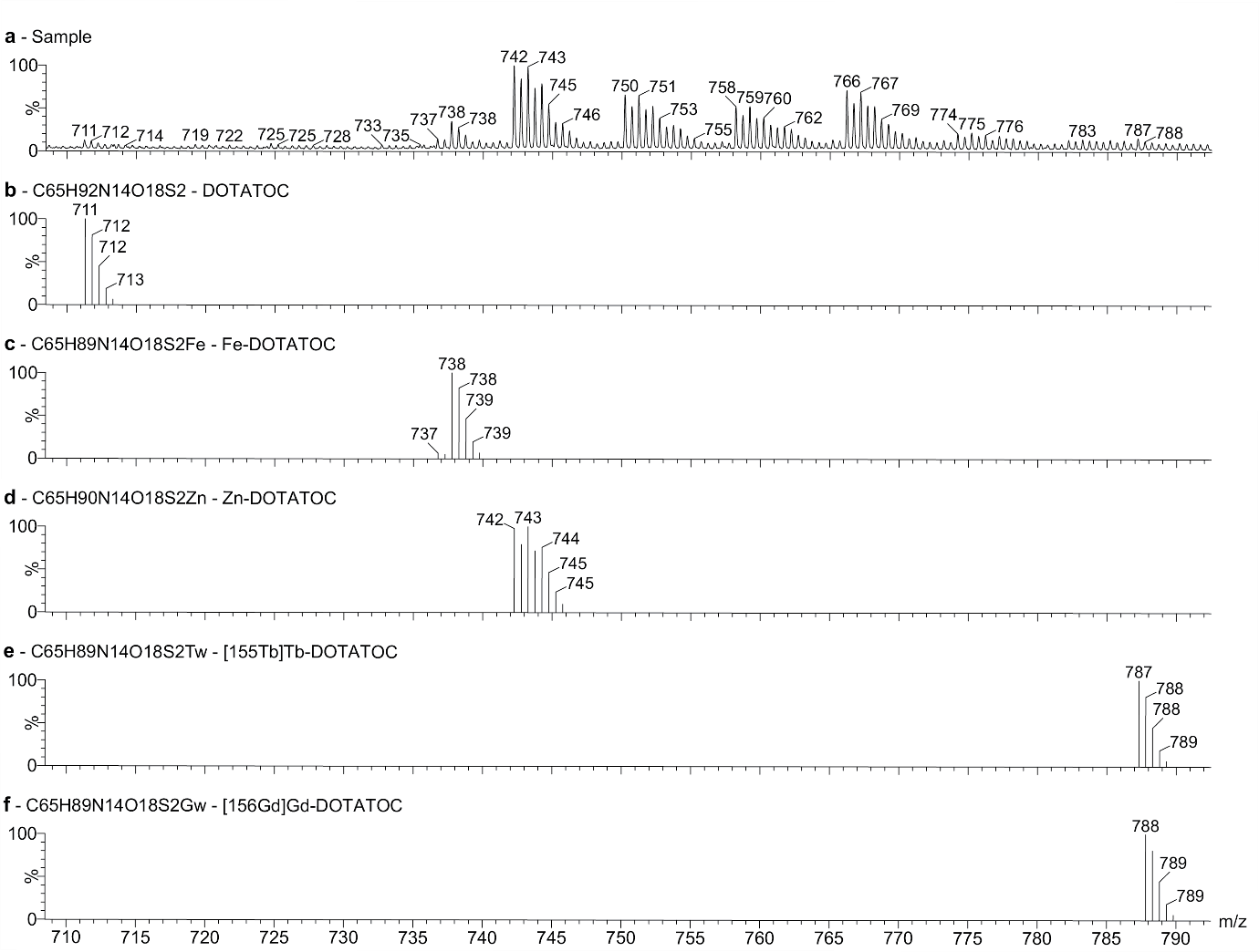


**Fig. S8** Representative mass spectrum of a [^155^Tb]Tb-DOTATOC radiolabeled sample with radiolabeling yield <95% (DOTATOC radiolabeled with terbium-155 from production 9 at 100 MBq/nmol) (**a**) in comparison with the theoretical mass spectra of free DOTATOC (**b**), Fe-DOTATOC (**c**), Zn-DOTATOC (**d**), [^155^Tb]Tb-DOTATOC (**e**) and [^156^Gd]Gd-DOTATOC (**f**). Mass peaks between 750 and 780 are oxidized species of Zn-DOTATOC.

1. **Recycling of the target material**

*Purpose:* The recycling process to recover the target material after the purification of terbium-155 was developed in order to be able to reuse [^155^Gd]Gd_2_O_3_ and [^156^Gd]Gd_2_O_3_ for future irradiations. The recycling process was performed with up to 100 mg of gadolinium per batch.

*Methods:* In the terbium-155 radiochemical separation process from the target material described in this work, terbium-155 was principally separated from gadolinium on a Sykam column using α-HIBA as eluent. The fraction eluted from Sykam column containing gadolinium was collected for recycling. This solution was stored for several months to let it decay, after which it was processed to recycle the target material. Firstly, the organic fraction deriving from the α-HIBA was removed from the solution by loading it onto an AG-MP50 cation exchange resin under moderately acidic conditions (HCl 1.0 M). After a column rinse with 0.1 M HNO_3_, the gadolinium fraction was eluted with ~20 mL concentrated 7.0 M HNO_3_. The eluted solution was then evaporated to dryness. The residue obtained was dissolved in 0.1 M NH_4_NO_3_ (pH 3.5) and loaded onto a Sykam column for purification using 0.13 M α-HIBA (pH 4.5) as eluent. The eluted volume was collected in 5 mL fractions, which were analyzed by means of γ-spectrometry. The fractions containing only long-lived radionuclides of gadolinium were collected and gadolinium was then separated from the α-HIBA with the use of an AG-MP50 resin column. In particular, after loading the gadolinium solution onto AG-MP50 resin in 1.0 M HCl and a column rinse with 0.1 M HNO_3_, gadolinium was eluted with ~20 mL concentrated 7.0 M HNO_3_. The purified enriched Gd nitrate in the quartz tube was then heated with a torch to decompose the nitrate to the oxide.

*Results:* Thanks to the natural presence of long-lived radionuclides in tracer amounts in the recycling solution, it was possible to define the elution profile of gadolinium and the other contaminants in the recycling solution from Sykam resin (Fig. S9). It was then possible to collect only the fractions containing gadolinium and use them to transform the gadolinium from nitrate to oxide, ready for reuse for future irradiations.

**
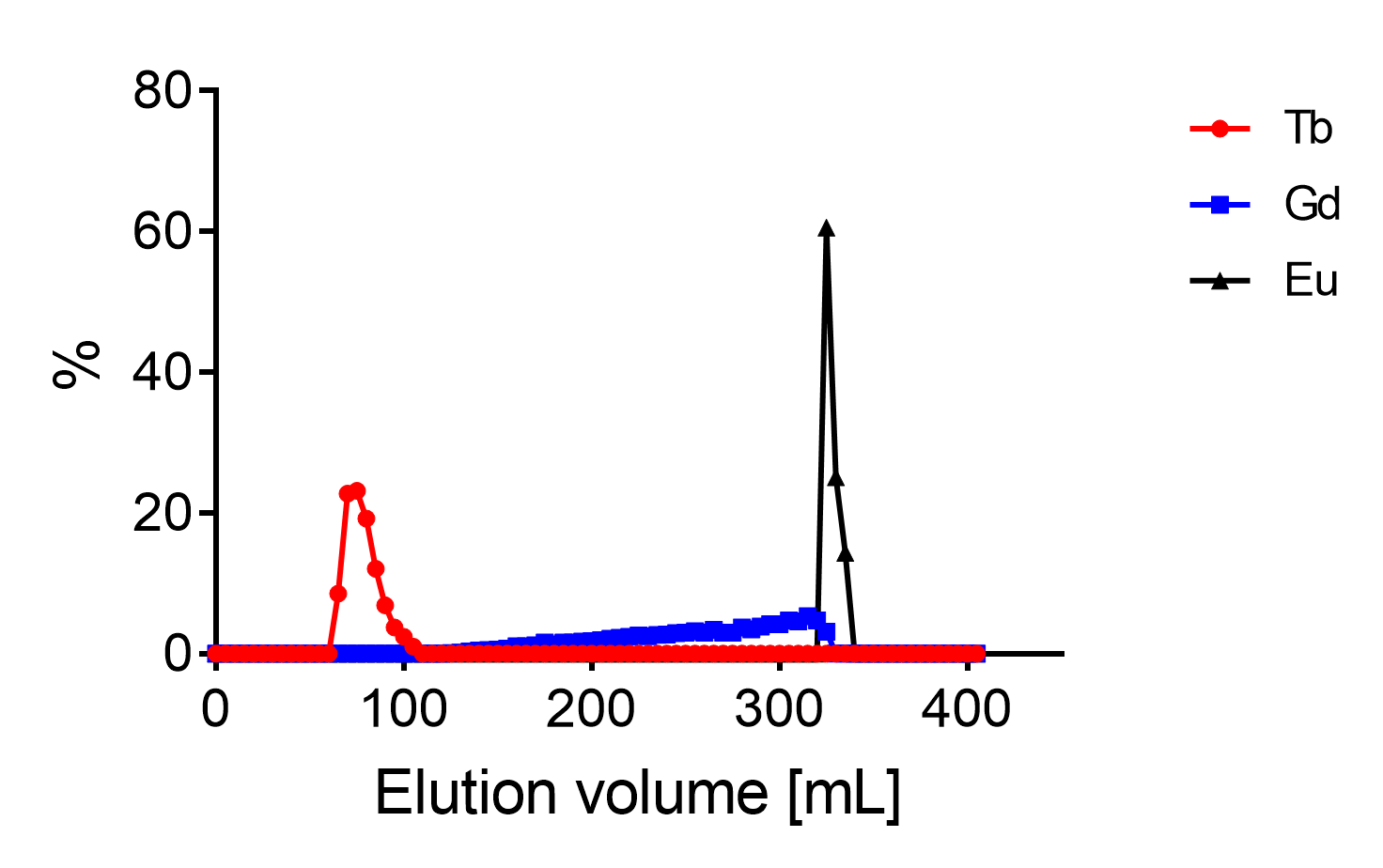
**

**Fig. S9** Elution profile of terbium, gadolinium and europium from Sykam resin during recycling process.

1. **In vitro experiments**

**Cell culture**

AR42J tumor cells (a somatostatin receptor (SSTR)-positive exocrine rat pancreatic cancer cell line, ECACC 93100618) (Hofsli et al. 2002) were purchased from Health Protection Agency Culture Collections (Salisbury, U.K.). The cells were cultured in RPMI-1640 cell culture medium supplemented with 20% fetal calf serum (FCS), l-glutamine and antibiotics.

**Cell uptake and internalization experiments**

*Purpose:* The aim of these in vitro studies was to verify if [^155^Tb]Tb-DOTATOC undergoes SSTR-specific cellular uptake and internalization in AR42J cells.

*Methods:* DOTATOC, dissolved in Milli-Q water at the concentration of 1 mM, was radiolabeled with terbium-155 following the same procedure used for the determination of the chemical purity, up to a molar activity of 20 MBq/nmol. The radiopeptide obtained at a radiochemical purity ≥99%, was readily diluted and used for the preclinical evaluation without further purification. Uptake and internalization studies were carried out according to a previously published procedure (Borgna et al. 2021). AR42J tumor cells were seeded in poly-L-lysine-coated 12-well-plates at the density of 1,000,000 cells/2 mL and grown overnight (37 °C, 5% CO_2_). [^155^Tb]Tb-DOTATOC, prepared at the molar activity of 20 MBq/nmol, was added (15 kBq, 25 μL, 0.75 pmol per well, diluted to 1 mL in RPMI-1640 containing 1% FCS, l-glutamine and antibiotics) to the cells. Some wells were co-incubated with 1 mL 0.1 mM DOTATOC to check for specific uptake. After incubation for 2 and 4 h at 37 °C, the cells were rinsed three times with 1 mL ice-cold PBS to determine the total uptake of the radiopeptide. The internalized fraction was determined in other wells where the cells were rinsed with 1 mL ice-cold PBS, incubated for 30 min with acidic stripping buffer (1 mL 0.05 M glycine stripping buffer in 100 mM NaCl, pH 2.8) followed by an additional rinsing step with ice-cold PBS. After those steps, the tumor cells were lysed by adding 1 mL NaOH (1 M) to each well. The cell suspensions were transferred to 4-mL tubes for measurement in a γ-counter (Perkin Elmer, Wallac Wizard 1480). For the estimation of the protein concentration, a Micro BCA Protein Assay (Pierce, Thermo Scientific) was performed. The measured radioactivity with the γ-counter was then standardized to the average content of protein in a single well. The experiments were performed in sextuplicates (n=1) and graphs were prepared using GraphPad Prism software (version 8).

*Results:* DOTATOC was successfully radiolabeled with terbium-155 at 20 MBq/nmol obtaining a radiolabeling yield > 95%. In vitro cell uptake and internalization studies using this radiopeptide showed specific uptake in SSTR-positive tumor cells (Fig. S9). Moreover, the results were comparable to previously published results obtained with the respective ^177^Lu-labeled versions (Borgna et al. 2021). The cell uptake and internalization of [^155^Tb]Tb-DOTATOC were 13 ± 1% and 11 ± 1% of total added activity, respectively, after 4 h (Fig. S10a). In cells blocked with an excess of DOTATOC, the uptake was <1% of the added activity (Fig. S10b).


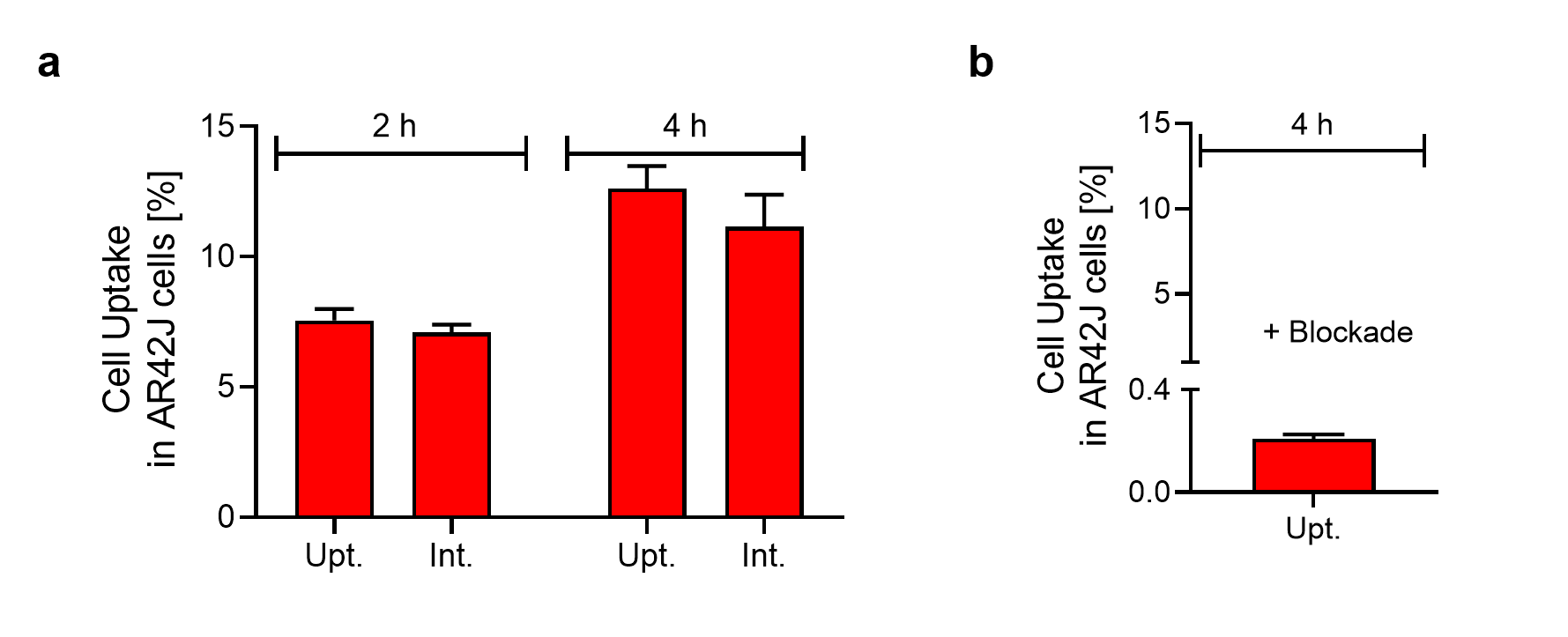


**Fig. S10** Cell uptake and internalization of [^155^Tb]Tb-DOTATOC in SSTR-positive AR42J tumor cells after incubation at 37°C (**a**). SSTR-specific binding is proven by experiments with pre-incubation of the cells with excess DOTATOC (**b**).

1. **In vivo studies**

All applicable international, national, and/or institutional guidelines for the care and use of laboratory animals, in particular, the guidelines of Swiss Regulations for Animal Welfare were applied. The SPECT/CT studies were ethically approved by the responsible Committee of Animal Experimentation and permitted by the responsible cantonal authorities (license N° 75721).

1. **SPECT/CT imaging studies**

A small-animal, 4-head multiplexing, multipinhole camera (NanoSPECT/CT; Mediso Medical Imaging Systems, Budapest, Hungary) was employed for SPECT/CT imaging studies, as previously reported (Borgna et al. 2021). Each head was outfitted with a tungsten-based aperture of nine 1.4-mm-diameter pinholes and a thickness of 10 mm. SPECT/CT images were acquired by use of Nucline software (version 1.02; Mediso, Ltd., Budapest, Hungary). The camera energy peaks were set at 46 keV (±8.5%), 86 keV (±8.5%) and 105 keV (±10%). After the acquisitions, SPECT data were reconstructed iteratively with HiSPECT software (version 1.4.3049; Scivis GmbH, Göttingen, Germany). The real-time CT reconstruction used a cone-beam filtered back-projection. CT scans were obtained using a tube voltage of 55 kVp and an exposure time of 1.0 s per view. SPECT and CT data were automatically co-registered because both modalities shared the same axis of rotation. The fused datasets were analyzed with the VivoQuant postprocessing software (version 3.5, inviCRO Imaging Services and Sofware, Boston, USA).

**References**

Asti M, Tegoni M, Farioli D, Iori M, Guidotti C, Cutler CS, et al. Influence of cations on the complexation yield of DOTATATE with yttrium and lutetium : a perspective study for enhancing the ^90^Y and ^177^Lu labeling conditions. Nucl. Med. Biol. 2012;39(4):509–17.

Borgna F, Barritt P, Grundler P V., Talip Z, Cohrs S, Zeevaart JR, et al. Simultaneous visualization of ^161^Tb-and ^177^Lu-Labeled somatostatin analogues using dual-isotope SPECT imaging. Pharmaceutics. 2021;13(4):1–13.

Gracheva N, Müller C, Talip Z, Heinitz S, Köster U, Zeevaart JR, et al. Production and characterization of no-carrier-added ^161^Tb as an alternative to the clinically-applied ^177^Lu for radionuclide therapy. EJNMMI Radiopharm. Chem. 2019;4(12):1–16.

Hofsli E, Thommesen L, Norsett K, Falkmer S, Syversen U, Sandvik AK, et al. Expression of chromogranin A and somatostatin receptors in pancreatic AR42J cells. Mol. Cell. Endocrinol. 2002;194(1–2):165–73.

Müller C, Reber J, Haller S, Dorrer H, Bernhardt P, Zhernosekov K, et al. Direct in vitro and in vivo comparison of ^161^Tb and ^177^Lu using a tumour-targeting folate conjugate. Eur. J. Nucl. Med. Mol. Imaging. 2014;41(3):476–85.

Nica N. Nuclear Data Sheets for A=155. Nucl. Data Sheets. Elsevier Inc.; 2019;160:1–404.

Reich CW. Nuclear Data Sheets for A = 154. Nucl. Data Sheets. Elsevier Inc.; 2009;110(10):2257–532.

Reich CW. Nuclear Data Sheets for A = 156. Nucl. Data Sheets. Elsevier Inc.; 2012;113(11):2537–840.
